# Competition response of cloud supersaturation explains diminished Twomey effect for smoky aerosol in the tropical Atlantic

**DOI:** 10.1073/pnas.2412247122

**Published:** 2025-03-24

**Authors:** Jeramy L. Dedrick, Christian N. Pelayo, Lynn M. Russell, Dan Lubin, Johannes Mülmenstädt, Mark Miller

**Affiliations:** ^a^Scripps Institution of Oceanography, University of California, San Diego, La Jolla, CA 92037; ^b^Atmospheric, Climate, and Earth Sciences Division, Pacific Northwest National Laboratory, Richland, WA 99354; ^c^Department of Environmental Sciences, Rutgers University, New Brunswick, NJ 08901

**Keywords:** aerosol, clouds, climate, supersaturation, Twomey

## Abstract

The Twomey effect brightens clouds by increasing aerosol concentrations, which activates more droplets and decreases cloud supersaturation in response to more competition for water vapor. To quantify this competition response, we used marine low cloud observations in clean and smoky conditions at Ascension Island in the tropical South Atlantic during the Layered Aerosol Smoke Interactions with Cloud (LASIC) campaign. These observations show similar increases in droplet number for increased accumulation-mode particles from surface-based and satellite cloud retrievals, demonstrating the importance of below-cloud aerosol measurements for retrieving aerosol–cloud interactions (ACI) in clean and smoky aerosol conditions. Four methods for estimating cloud supersaturation from aerosol–cloud measurements were compared, with cloud scene-based and parcel-based methods showing sufficient variability for a strong dependence on both aerosol accumulation number concentration and cloud-base updraft velocities. Decomposing aerosol-related changes in cloud albedo and optical depth shows the calculated competition response accounts for dampening the activation response by 12 to 35%, explaining the diminished Twomey effect at high aerosol concentrations observed for smoky conditions at LASIC and previously around the world. This result was consistent for independent supersaturation retrievals by cloud scene-based droplet number and cloud condensation nuclei and parcel-based multimode size-resolving Lagrangian methods. Translating aerosol effects to local radiative forcing with clean conditions as a proxy for preindustrial and smoky conditions for present-day showed that the competition response reduces cooling from the Twomey radiative forcing by 12 to 35%, providing an essential process-specific constraint for improving the representation of aerosol competition in climate model simulation of indirect aerosol forcing.

## Significance

Aerosol–cloud interactions (ACI) are one of the most uncertain aspects of global climate predictions, in part because there are insufficient process-specific constraints from observations. This method of decomposing the most radiatively important impact of aerosols on clouds known as the Twomey effect incorporates retrieved supersaturation from two independent sets of observations to constrain the feedback of aerosol particles on cloud properties. The method quantifies the reduction of the Twomey effect at high aerosol concentrations. While previously observed, this diminishing of the Twomey effect has never been explained quantitatively by observations. In addition, the results provide the direct observational constraints on a parcel-based approach that is embedded in many climate models.

Low-level marine clouds exert a strong cooling on the planet by reflecting incoming solar radiation and thus play an important role in the global radiative budget (1). Responses of cloud microphysical and macrophysical properties to changes in aerosol, known as aerosol–cloud interactions (ACI), have such large uncertainty in the magnitude of cooling (2, 3) that climate projections provide only low confidence (4). Quantifying the man-made contribution to this cloud cooling requires distinguishing preindustrial and present-day ACI and leads to the uncertainty in anthropogenic radiative effects in global climate models (5, 6) because of the complexities of cloud droplet activation dependence on particle size, concentration, composition, cloud type, and region (7–10).

To evaluate this anthropogenic change in ACI requires finding present-day conditions that resemble preindustrial (11), but the paucity of suitably “pristine” conditions that represent preindustrial aerosol concentrations in the present-day atmosphere makes this approach challenging (5, 6, 12, 13). Satellite retrievals of aerosol and cloud properties provide increased spatial and temporal coverage but are limited for mixed aerosol–cloud scenes that rely on aerosol retrieved adjacent to clouds (14, 15) or on very clean conditions with high uncertainties (6, 16). Open ocean regions of the South Pacific, South Atlantic, and Southern Ocean may be the few remaining areas that represent preindustrial conditions because they are characterized by persistent low number concentrations of natural, mostly marine, cloud condensation nuclei, and limited anthropogenic aerosol from continents (11, 17, 18). The most immediate effect of an anthropogenic increase in aerosol emissions is an increase in cloud droplet number concentration and concomitant reduction in cloud droplet size leading to an increased cloud albedo (3), which is known as the “Twomey effect” (19). Differences in cloud structure can mask this effect showing weak-to-no sensitivity between cloud droplet number and marine biogenic aerosol in some regions (20, 21) but strong or competing sensitivities in others (22–24).

The Twomey effect is driven by increased aerosol particle number concentrations activating to more cloud droplets at approximately constant liquid water, where this “activation response” has been observed to be approximately linear at particle concentrations below 300 to 1,000 cm^−3^ but then the effect diminishes, becoming asymptotic at higher concentrations (19, 25, 26). This reduced effect at higher droplet concentrations has been explained by parcel models as attributable to the competition among the increased number of activating droplets for condensing water vapor. This competition reduces the maximum supersaturation reached in the cloud, which then dampens the droplet number increase and reduces the Twomey effect (7, 25–29). However, since cloud supersaturation has never been measured directly and varies with both aerosol concentrations and cloud updraft velocities (9, 30), parcel models have been the only tool available to untangle the role of supersaturation. The limitations of parcel models are evident in the variety of observations in the North Atlantic (31), Northwest Pacific (32), and Northeast Pacific (33) that report ACI that differ from the reported parcel model simulations.

For global climate models that parameterize droplet activation with the multimode size-resolving Lagrangian approach (34), the model-simulated vertical velocity and aerosol properties used to represent the competition effect may not sufficiently represent observations. The small-scale fluctuations in vertical velocity and humidity are parameterized at model grid scales using different approaches (35, 36) that cannot fully represent the subgrid scale variability. These simplified representations of aerosols and updraft velocities are needed at global scales for efficiency, but comparisons to observations are needed to assess the associated uncertainty in the Twomey effect. The competition between activated particles for available supersaturation is computed in climate model parameterizations (30, 37), but an observational constraint for this process requires cloud-base updraft velocity retrievals and composition-resolved aerosol size distributions. Given the difficulty of retrieving these important geophysical variables from satellite (38, 39), surface-based retrievals, while sporadically collected, add essential complementary information to satellite assessments.

Cooling results from the Twomey effect as well as from covariability of meteorological responses with aerosol loading and is quantified as the radiative forcing by aerosol–cloud interactions (RF_ACI_). Global estimates of RF_ACI_ range from −2.51 to −0.18 W m^−2^ in simulations of global climate models (2, 3, 16–18, 40–45) (*SI Appendix*, Table S1). To constrain this type of model simulation with observations, contrasting clean and polluted conditions can be used as an opportunistic experiment to provide an analog of the preindustrial to present-day forcing (46). McComiskey et al. (47) calculated such an analog of radiative forcing using surface-based observations as a proxy, estimating a much higher effect for cloudy conditions of −19.0 to −4.3 W m^−2^ for the northern California coast. However, few similar constraints are available for other regions, and the range of aerosol concentrations and the prevalent cloud structure affect the representation of both preindustrial and present-day conditions.

## Results and Discussion

To evaluate the dependence of the Twomey effect in both natural and perturbed aerosol conditions, we retrieved supersaturation, aerosol and droplet concentrations, and radiative forcings from surface-based measurements collected in the remote tropical South Atlantic boundary layer in 2016–2017 during the Department of Energy (DOE) Atmospheric Radiation Measurement (ARM) Layered Aerosol Smoke Interactions with Cloud (LASIC) campaign (48). The continuous 18-month record of observations shows the cloud droplet properties for aerosol conditions ranging from those similar to preindustrial (because of the remoteness of the location) to those more similar to present day (because of substantial transport of seasonal smoke), both superimposed on boundary-layer cloud conditions that remain fairly consistent year-round. Comparing these observations to simulated aerosol and updraft regimes provides context for the observed range of droplet size and number concentration for the conditions measured. The retrieval of three observation-based estimates of supersaturation compared to a commonly used model parameterization provides an apportionment of cloud albedo and optical depth changes. This apportionment separates the “activation response” of the number of aerosol particles activated from what we propose be called the “competition response” of the cloud supersaturation to the growing droplets (in addition to the “dynamical response” of the updraft to the aerosol). These observations were used to calculate the analog of RF_ACI_ for the low-level marine clouds over Ascension Island for constraining global simulations.

### Observed Accumulation-Mode Aerosol Increases with Retrieved Cloud Droplet Number.

**Fig. 1.**
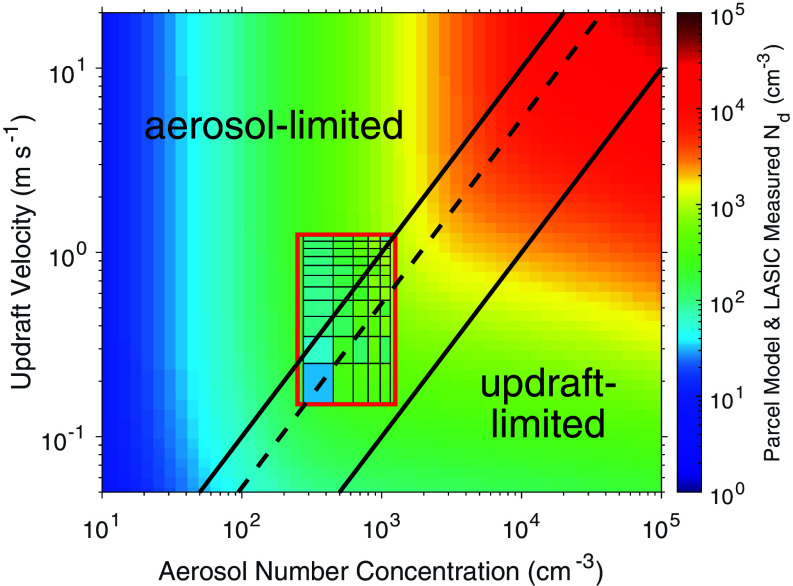
Joint histogram of updraft velocity and aerosol number concentration on cloud droplet number concentration (N_d_) evaluated using air parcel models (7, 27) and LASIC measurements (shown in inset grid). Solid lines delineate “aerosol-limited,” “transitional,” and “updraft-limited” regimes defined by (27). The dashed line delineates aerosol- and updraft-limited regimes defined by (7).

Ascension Island was covered by cloud fractions greater than 75% for more than 60% of the time during LASIC (25). Single-layer low clouds occurred approximately 76% of the time during the nonbiomass burning season (November to May) and 81% of the time during the biomass burning season (June to October), with no significant difference between clean (83%) and smoky (80%) aerosol conditions. For 17 mo of LASIC observations, 37% of clean and smoky 2 h periods (*n* = 1,573) were identified as overcast, single-layer, low-cloud scenes with 20 to 300 g m^−2^ liquid water path (LWP) and well-mixed boundary layers (*SI Appendix*, *Text S1*). The average cloud droplet number concentration doubled from 106 ± 87 cm^−3^ to 213 ± 195 cm^−3^, and effective radius decreased from 14 ± 4 µm to 11 ± 4 µm between clean and smoky conditions (*SI Appendix*, Fig. S1). Placing these observations in a theoretical context with an aerosol parcel model (7) shows the dependence of cloud droplet number concentration on updraft velocity and aerosol number concentration (Fig. 1). LASIC covers a smaller range of aerosol number and updraft velocity than that of the parcel model (Fig. 1), but their overlap with the simulated joint histogram shows that the majority, 88% (27) to 95% (7), of observations are classified as “aerosol-limited” (insensitive to updraft) and the remainder as either “transitional” (12%) (27) or “updraft-limited” (5%) (7) in the modeled parameter space. The similarity of the colors for the observed and simulated droplet concentrations shows the range of predicted values to be quite consistent with the retrieved values.

**Fig. 2.**
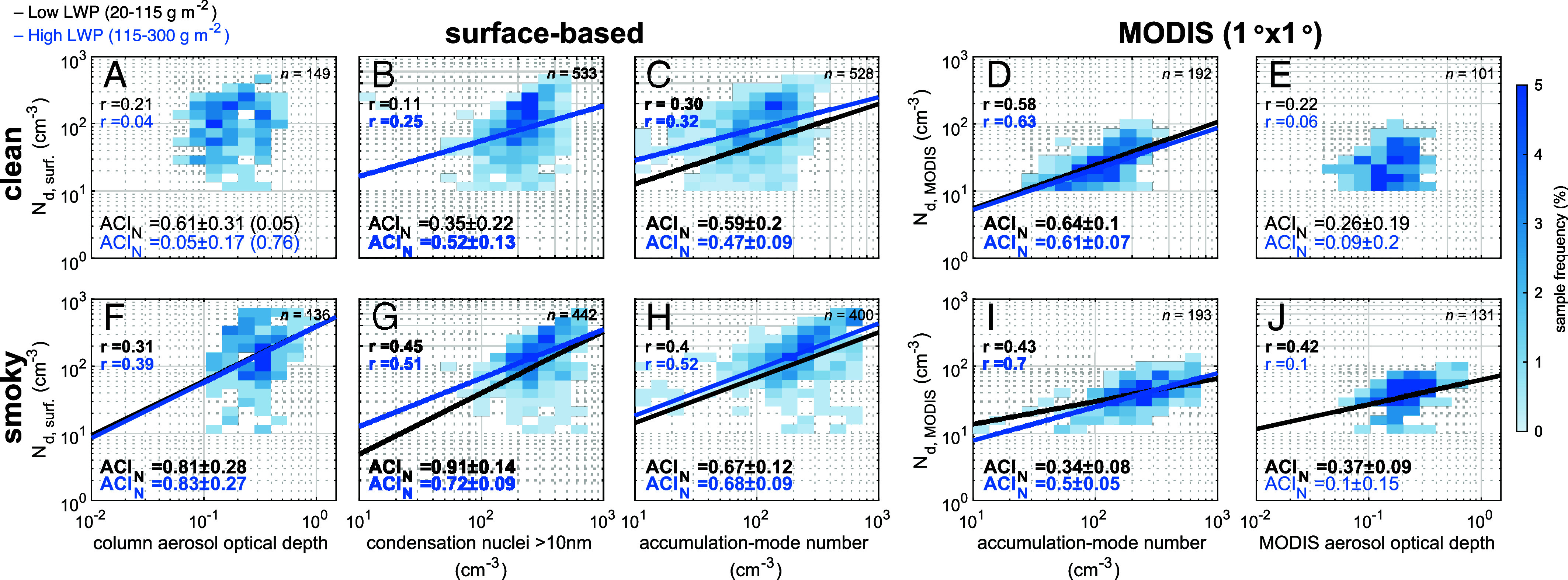
Joint histograms of cloud droplet number concentration (N_d_) versus aerosol proxies (ACI_N_) in clean (*A*-*E*) and smoky conditions (*F*-*J*). Surface-based droplet number and satellite-retrieved droplet number from the Moderate Resolution Imaging Spectroradiometer (MODIS) are compared to surface-based and satellite aerosol metrics. Color scale represents the percent of sample points in each bin. The number of sample points (*n*) in each regression are shown at the top right of each panel. ACI_N_ is computed as log–log slope and SE (±) from linear regression. Regression fits shown in bold and as lines for statistically significant (*P* < 0.05, two-tailed *t* test) fits that were at least weakly correlated (r > 0.25). ACI_N_ separated by low (20 to 115 g m^−2^) (black line fit) and high (115 to 300 g m^−2^) (blue line fit) liquid water path (LWP).

ACI indices (ACI_N_) represent the logarithmic change in cloud droplet number concentration (*Methods*) relative to aerosol properties at constant LWP (Fig. 2) and have been reported for a range of different surface-based and satellite aerosol proxies (6, 22, 23, 47, 49, 50) (*SI Appendix*, Table S2). For the observed clean conditions, the strongest correlation between surface-based aerosol measurements and cloud droplet number retrievals was for accumulation-mode (~150 nm) number concentration as the aerosol proxy (r = 0.3 to 0.32, *P* < 0.05 using a two-tailed *t* test) with ACI_N_ of 0.47 ± 0.09 and 0.59 ± 0.20 (Fig. 2*C*). During smoky conditions, surface-based cloud droplet number retrievals similarly correlated with column aerosol optical depth and condensation nuclei (>10 nm) number concentrations (r = 0.31 to 0.51) (Fig. 2 *F* and *G*) as the surface-based accumulation-mode number (r = 0.41 to 0.52) (Fig. 2*H*), but the accumulation-mode number ACI_N_ was lower (ACI_N_ = 0.67 to 0.68) than that when the other two proxies were used (ACI_N_ = 0.72 to 0.91). In clean conditions, these differences can be explained by column aerosol optical depth being based on aerosol mass rather than number concentration and by condensation nuclei (>10 nm) number being weighted by small particles that are too small to activate to droplets (25), making both poor proxies for cloud condensation nuclei. Aitken-mode (~40 nm) number concentration was also weakly correlated (r < 0.2) with low ACI_N_ (<0.1) (*SI Appendix*, Fig. S2 *B*, *E*, *G*, and *J*). The higher magnitudes of ACI_N_ using the column aerosol optical depth and condensation nuclei number aerosol proxies during smoky marine conditions may illustrate a “shallowing effect” of the microphysical responses when comparing clean and smoky aerosol conditions due to aerosol retrieval uncertainties that are particularly sensitive to aerosol optical properties in clean conditions (6, 16).

The observed clean marine accumulation-mode ACI_N_ of 0.47 to 0.59 was similar to the value of 0.3 ± 0.21 reported for Ascension Island low clouds (49), as well as to surface and aircraft measurements for clean marine stratocumulus of 0.2 to 0.7 in the Northeast Atlantic (51), of 0.18 to 0.9 in the Northeast Pacific (22, 47, 52), and of 0.32 to 0.48 in the Arctic (24) (*SI Appendix*, Table S2). Accumulation-mode ACI_N_ during smoky conditions (0.67 ± 0.10) were slightly higher than those observed during clean conditions (Fig. 2*H*) and were consistent with previous surface-based and in-cloud estimates in the range of 0.21 to 0.62 for polluted marine conditions (32, 53) (*SI Appendix*, Table S2).

The satellite-retrieved aerosol optical depth only correlated (r = 0.42) with satellite-retrieved droplet number in smoky conditions at low LWP (20 to 115 g m^−2^) (Fig. 2 *E* and *J*). Because of the challenges retrieving aerosol in cloudy scenes, particularly the use of satellite-retrieved aerosol optical depth at the edge of cloud scenes and satellite optical depth retrieval uncertainty in very clean conditions (6), the low ACI_N_ and correlation for aerosol optical depth was expected. However, the surface-based accumulation-mode number yielded well-correlated ACI_N_ (r = 0.58 to 0.63) with satellite-retrieved droplet number (Fig. 2*D*) and effective radius (*SI Appendix*, Fig. S3) that were very similar in magnitude to using surface-based retrievals in clean and smoky conditions, with similar results for clean condensation nuclei for high LWP (115 to 300 g m^−2^) clouds (*SI Appendix*, Fig. S3). The weaker correlations for surface-based N_d_ retrievals in Fig. 2 *C* and *H* compared to satellite retrievals in Fig. 2 *D* and *I* are likely attributable to the larger variability in aerosol, updraft, supersaturation, and LWP represented by the more frequent (twice hourly) and more localized surface-based measurements compared to the spatial averaging of cloud fields available from satellite. These combined results show that accumulation-mode aerosol and cloud droplet number concentration retrieved at the surface-based describe cloud droplet number concentration changes in clean and smoky conditions that yield similar ACI_N_ to those retrieved by MODIS.

### Cloud Supersaturations Control ACI.

**Fig. 3.**
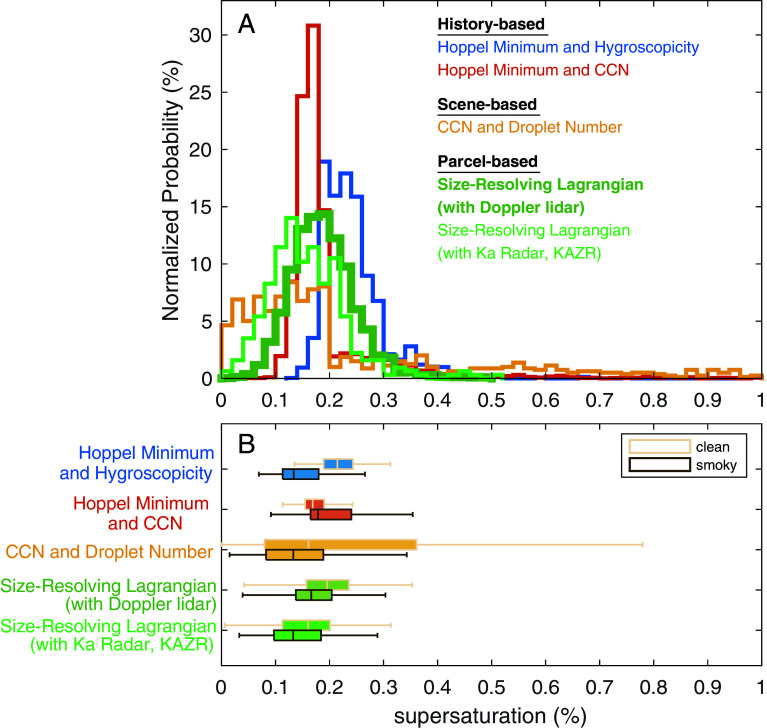
Histograms of the history, scene- and parcel-based multimode supersaturations (*A*) retrieved from aerosol size distribution Hoppel minima, cloud condensation nuclei (CCN), composition-based hygroscopicity, radiometric droplet number, and updraft measurements (Doppler lidar and Ka-band Radar, KAZR) and their comparison for clean (beige) and smoky (brown) conditions (*B*). Boxes and center line represent the 25th/75th percentiles and median, respectively. Whiskers represent 10 to 90th percentiles.

Cloud supersaturation is the fundamental property that controls aerosol effects on cloud microphysics as it determines the smallest particles to be activated to cloud droplets, but because it cannot be measured directly, its role in ACI has not been quantified. Improving models requires separating ACI processes to distinguish between accurate simulations and those that appear to match observations but for the wrong reasons (12, 54). Two history-based, one scene-based, and one parcel-based approaches provide estimates of observationally constrained supersaturation (*SI Appendix*, *Text S3*). The two history-based methods utilize measurements that are affected by ensembles of cloud updraft velocities to obtain “Hoppel minima”(25) retrieved from surface-based aerosol size distributions to calculate the maximum effective supersaturation associated with the observed particle growth from the Aitken to the accumulation mode by cloud processing. The scene-based method compares CCN spectra with the radiometer-retrieved cloud droplet distributions, which are based on 2-h averaged individual cloud scenes. These two history-based and one scene-based observational methods yield similar statistical distributions with median values of 0.15 to 0.25% (Fig. 3*A*) but the supersaturations retrieved for each method are only weakly correlated with each other (r = 0.15 to 0.31; *SI Appendix*, Fig. S4). These aerosol observations provide consistent representations of effective cloud supersaturations that are well within supersaturations <0.3% for cloud-base updraft velocities <1 m s^−1^ as reported for marine cumulus and stratocumulus (55–57) but substantially lower than recent global estimates of >0.5% (58). The parcel-based estimate of supersaturation was the Multi-Mode Size-Resolving Lagrangian method, which is a parameterization commonly used in global models for multimodal aerosol distributions (34, 59). The supersaturations estimated from this method were 0.19 ± 0.06% and 0.16 ± 0.09% using Doppler lidar and Ka-band radar updraft velocities, respectively (Fig. 3 *A* and *B*). For comparison to a standard calculation of supersaturation, the quasisteady state approximation (60) was also computed (*SI Appendix*, *Text S3* and Fig. S5) but retrieved lower supersaturations (0.076 ± 0.09%, *SI Appendix*, Fig. S5) than the other four methods because it does not represent the activation of aerosol near cloud base or the maximum supersaturation.

The parcel-based method is included for comparison to parameterizations in climate and regional models that nominally resolve the competition response based on multimodal aerosol competition for water vapor and the updraft velocity, although the fidelity of its representation may be limited based on the assumptions employed. The parcel-based approach considers the average cloudbase updraft velocities and neglects smaller-scale turbulent fluctuations that can impact activation near cloud base (61, 62).

Clean marine clouds had higher mean supersaturations than smoky clouds for all but the history-based Hoppel Minimum and CCN estimates (which had no distinguishable difference, Fig. 3*A*), with the largest median difference between conditions, relative to SD, evident in comparing clean cloud supersaturations of 0.22 ± 0.06% to smoky supersaturations of 0.17 ± 0.06% (Fig. 3*B*). This mean supersaturation difference was statistically significant (*P* < 0.01, two-sample *t* test) and shows the effect of higher number concentrations of smoke aerosol particles competing to take up water faster and deplete cloud supersaturation sooner, thereby decreasing the maximum supersaturation reached.

The history-based effective supersaturations have a very weak correlation to the measured updraft velocities (r < 0.2; *SI Appendix*, Fig. S8), in contrast to studies that used parcel-like observations from aircraft and found moderate negative correlations (r ~ −0.5) (9, 55, 56). Likely this difference results from the averaging inherent in the history-based retrievals, namely that each observed value results from an ensemble history of cloud updraft velocities that processed the aerosol size distribution to produce the Hoppel minimum. The scene-based CCN and Droplet Number method also represents an ensemble of parcel updraft velocities that contributed to the cloud scene averaged droplet number and size, but the 2-h averaging time is sufficient to preserve some variability among parcels. Because this limitation means that history-based methods cannot retrieve the parcel-to-parcel dependence on updraft, the scene-based (CCN and Droplet Number) and Multi-Mode Size-Resolving Lagrangian Parcel (using measured updraft velocity from Doppler lidar) methods were used. These methods apportioned and compared two independent observation-constrained changes in cloud properties to the aerosol and updraft conditions during LASIC (*SI Appendix*, Fig. S9–S11). These two supersaturation estimates have weak to moderate, but statistically significant, correlations to droplet number and effective radius and sufficient range of updraft for a regression (r = 0.2 to 0.6; *SI Appendix*, Fig. S6).

### Competition Response Diminishes Aerosol Effects on Cloud Albedo, Optical Depth, and Radiative Forcing.

**Fig. 4.**
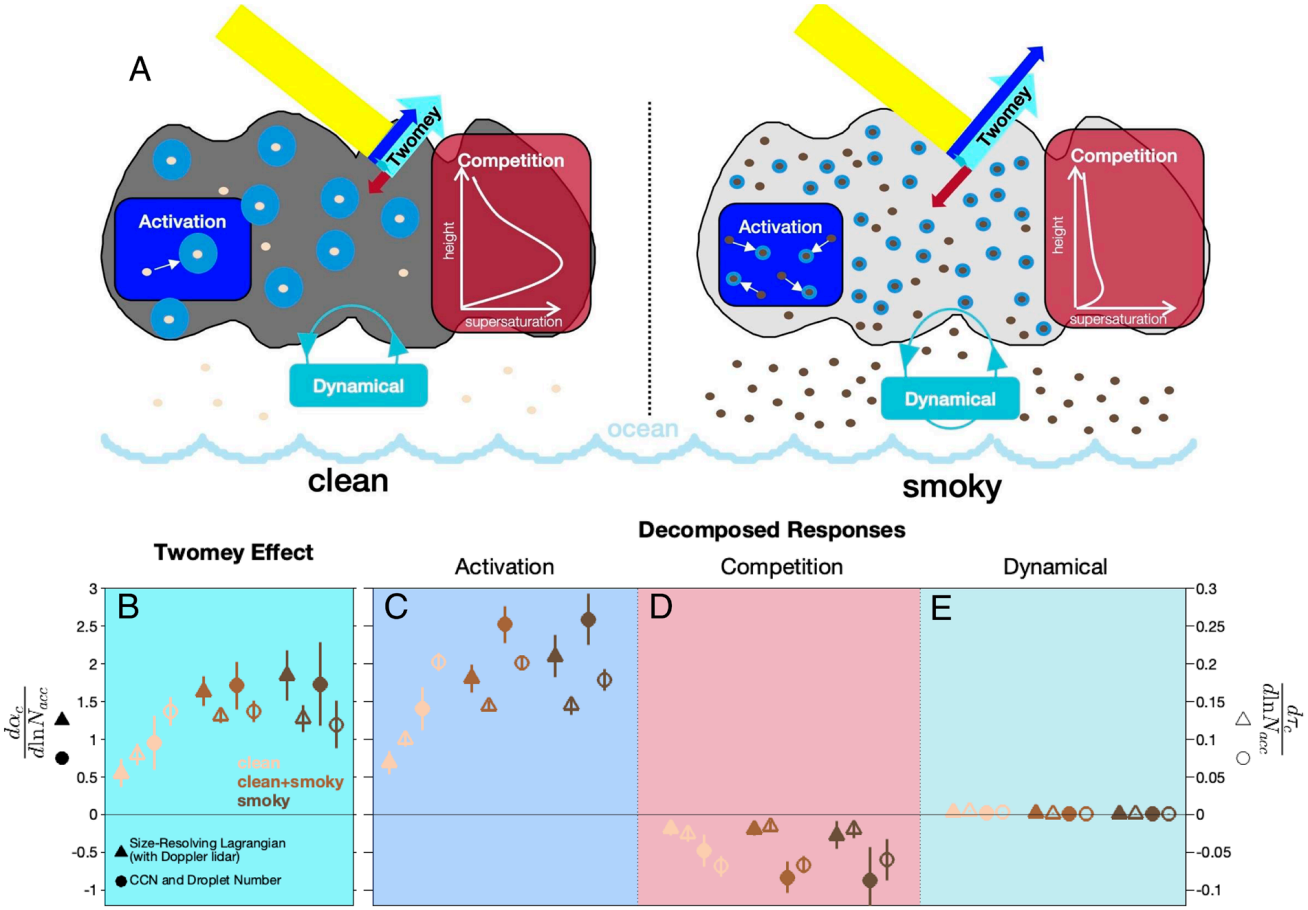
The Twomey effect and decomposed responses for cloud albedo and optical depth. (*A*) Schematic figure of Twomey responses (activation, competition, and dynamical) in clean and smoky marine clouds. Reflected arrows (not to scale) show outgoing shortwave changes of the three responses as well as the net Twomey effect. (*B*) Twomey effect of cloud albedo ($\alpha_{c}$) (filled symbols, left ordinate) and optical depth ($\tau_{c}$) (open symbols, right ordinate) to accumulation-mode number concentration (*N*_acc_) using Multi-Mode Size-Resolving Lagrangian with Doppler lidar (triangle) and CCN and Droplet Number (circle) supersaturations. (*C*–*E*) Twomey effect decomposed into activation (*C*), competition (*D*), and dynamical (*E*) responses. Symbols are colored by clean (beige), smoky (dark brown), and combined (clean + smoky; light brown) marine conditions. Whiskers represent SEM.

Evaluating the dependence of cloud albedo and optical depth on accumulation-mode aerosol concentration shows the Twomey effect on cloud albedo (Fig. 4*B* and *SI Appendix*, Table S4) ranges from 0.55 (for clean) to 1.84 (smoky), similar to the low end of the 1 to 4 range for mean Twomey effect observed in the eastern North Atlantic (63). Separating this effect into its governing processes (*Methods*) shows the activation response of nucleating more particles is the largest contribution, with this droplet number increase being dampened by the competition response of the additional particles taking up water more quickly and negligible enhancement from the dynamical response of particle-enhanced updraft velocity. These impacts on the net Twomey effect are shown by cloud albedo dependence on the droplet activation response generally increasing from clean to smoky conditions (Fig. 4*C*), with the albedo change in combined (clean + smoky) conditions (1.81 to 2.53) being similar to smoky (2.10 to 2.59) (*SI Appendix*, Table S4). The competition response (Fig. 4*D*) diminishes the net activation response to the Twomey effect by 12% using the parcel-based supersaturation (Size-Resolving Lagrangian with Doppler Lidar) and by up to 35% using the scene-based (CCN and Droplet Number) supersaturation. The competition response increases from clean (−0.48 to −0.18) to combined (−0.83 to −0.19) to smoky (−0.87 to −0.27) conditions, with the scene-based CCN and Droplet Number method giving responses more than twice the magnitude of the Size-Resolving Lagrangian method. This increase in competition response with smoky conditions illustrates how increases in aerosol number concentrations are further dampened by the competition response, which decreases cloud supersaturation (Fig. 4*B*). The consistent result between the methods provides two independent sources of evidence of the competition response on supersaturation, with the scene-based supersaturation showing a greater competition response to aerosol than the parcel-based supersaturation. The dynamical response (Fig. 4*E*) is negligible (0.01 to 0.03) at this location. Cloud optical depth responses (Fig. 4 *B*–*E*) are consistent with albedo but of smaller relative magnitude.

**Fig. 5.**
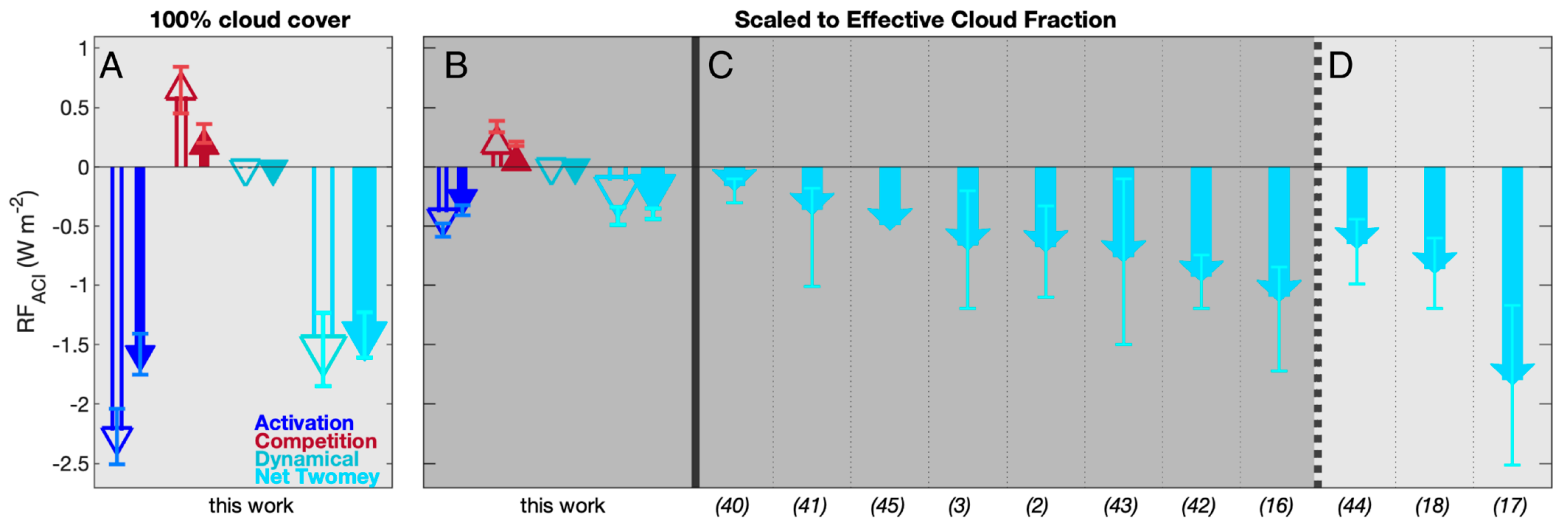
Radiative forcing by aerosol–cloud interactions (RF_ACI_) for 100% cloud cover and scaled to global-mean effective cloud fraction. (*A*) Local RF_ACI_ (100% cloud cover) calculated from LASIC measurements separated by Twomey responses and net Twomey effect. (*B*) LASIC RF_ACI_ scaled to global-mean values using an effective cloud fraction of 0.24 (30). RF_ACI_ in (*A*) and (*B*) computed using Multi-Mode Size-Resolving Lagrangian with Doppler lidar (filled arrows) and CCN and Droplet Number (unfilled arrows) supersaturation. RF_ACI_ from prior work (cited at *Bottom*) using (*C*) model-simulated aerosol and (*D*) regional aerosol. Arrows indicate the mean and direction of forcing (*Down*/negative: cooling, *Up*/positive: warming) colored by the response legend in (*A*). Whiskers on arrows represent the range (*Lower*/*Upper* bounds or one SD of the mean).

To facilitate comparisons of the retrieved Twomey effect to those from climate models, the combined clean and smoky effects on albedo were used to represent changes from preindustrial to anthropogenic aerosol conditions in a LASIC-based ACI radiative forcing (RF_ACI_), with the observed range of aerosol properties serving as a proxy for the anthropogenic perturbation to aerosols, following the approach of satellite-based investigations (*SI Appendix*, *Text S5*) (2, 40, 50). While LASIC measurements represent specific cloud and dynamic conditions, the LASIC RF_ACI_ provides a basis for comparing global and regional RF_ACI_ for overcast cloud conditions (Fig. 5*A*) and the mean global cloud field when scaled to the global mean effective cloud fraction for low marine clouds (2, 64) (Fig. 5*B*). The net RF_ACI_ (sum of Twomey responses) estimated from LASIC measurements (Fig. 5*A*) was −1.57 ± 0.19 W m^−2^ and −1.64 ± 0.31 W m^−2^, for the parcel and scene-based supersaturation approaches, respectively, indicating a cooling by reflectance of solar radiation. These 100% cloud cover LASIC RF_ACI_ can be scaled by an effective cloud fraction of 0.24 (median of 0.19 to 0.29 range) (2) to give −0.38 ± 0.046 W m^−2^ and −0.39 ± 0.074 W m^−2^ (Fig. 5*B*) for comparison to global observationally based Twomey forcing estimates (−1.72 to −0.18 W m^−2^; Fig. 5*C* and *SI Appendix,* Table S1), noting that the LASIC RF_ACI_ is not expected to be globally applicable. The scaled LASIC results are consistent with RF_ACI_ constrained with model-simulated aerosol (40, 41), suggesting that the strong seasonal perturbation by smoke aerosol (accumulation-mode number > 300 cm^−3^) in the otherwise very clean marine environment (accumulation-mode number < 120 cm^−3^) may be a reasonable proxy for changes from preindustrial conditions to present, even though they are lower than more regionally based aerosol estimates extrapolated to global RF_ACI_ (−2.51 to −0.44 W m^−2^; Fig. 5*D* and *SI Appendix*, Table S1) (17, 18, 44).

Consistent with the cloud albedo and optical depth effects, the competition response significantly decreases the activation response in the net Twomey RF_ACI_ (Fig. 5*A* and *SI Appendix*, Table S1). The Twomey RF_ACI_ during LASIC is consistent with studies showing smaller, more numerous droplets result in greater cloud reflectance (Fig. 5 *A* and *B*) (22, 24, 65, 66). The low dynamical contribution is consistent with the expectation that local meteorological effects are not sensitive to aerosol concentration for LASIC. The activation response is strongly negative at −1.73 to −2.42 W m^−2^ causing the negative RF_ACI_ (Fig. 5*A*). The competition response reduces RF_ACI_ by −0.19 ± 0.08 W m^−2^ (parcel) and 0.80 ± 0.19 W m^−2^ (scene) for a 12 to 35% reduction of cooling. The dynamical response is negligibly positive (−0.02 ± 0.00 W m^−2^).

### Implications for Climate Models.

As a constraint on aerosol effects on global climate, the remoteness of the LASIC marine boundary layer observations in the tropical South Atlantic provided clean and smoky conditions that serve, respectively, as proxies for preindustrial conditions with effectively only natural marine emissions and present-day conditions with substantial anthropogenic burning and combustion emissions. The decomposed responses of the cloud albedo and optical depth for the Twomey effect explain the significant dampening by the competition response of aerosol activation changes, whereas the dynamical response was too weak to meaningfully enhance the cloud albedo and optical depth (Fig. 4*A*). The resulting RF_ACI_ for the Twomey effect continued to imply cooling even after the 12 to 35% reduction by the competition response. This important response of supersaturation to droplet competition for water vapor in the Twomey effect decreases the modification by aerosol of cloud albedo and optical depth, and subsequently of radiative forcing. Isolating the competition response explains the diminishing of the Twomey effect at high aerosol concentrations. Because surface-based observations can provide cloud-base updraft velocities and composition-resolved aerosol size distributions that are not available from satellite, this work provides a more quantitative constraint that allows a more effective, process-specific diagnostic for evaluation of aerosol indirect forcings in global climate predictions.

## Methods

Between May 2016 and October 2017 (48), LASIC aerosol measurements were sampled on Ascension Island, St. Helena, in the remote tropical South Atlantic (8°S, 14°W; 360 m ASL) from a capped vertical inlet 10 m above ground level while radiometric instruments for microphysical retrievals were operated at a supplemental site at Wideawake Airfield (80 m ASL), 4 km away from the aerosol measurement site (*SI Appendix*, *Text S1*). ACI were computed as the log–log slope of the linearly regressed microphysical parameters (effective radius r_e,_ droplet number concentration N_d_) versus aerosol proxies (denoted as α) (47):

[1] $${\mathrm{ACI}}_{r}={\left.-\frac{\partial \ln r_{e}}{\partial \ln\alpha }\right|}_{\mathrm{LWP}},$$

[2] $${\mathrm{ACI}}_{N}={\left.\frac{\partial \ln N_{d}}{\partial \ln\alpha }\right|}_{\mathrm{LWP}}.$$

ACI_r_ was computed following a constant LWP assumption (19). ACI_N_ was similarly computed as ${\left.\frac{\partial lnN_{d}}{\partial ln\alpha }\right|}_{\mathrm{LWP}}$, which contains additional uncertainty of up to 78% because of the necessary approximations for N_d_ (67) related to the LWP, cloud fraction, and spatial and temporal averaging.

Following Varble et al. (63) and Quaas et al. (40), the sensitivity of cloud albedo ($\alpha_{c}$) to accumulation-mode concentration (*N*_acc_), $\frac{d\alpha_{c}}{dlnN_{acc}}$, represents the Twomey effect and can be decomposed into process-specific responses. Cloud albedo is described by the functional form α_c_ = α_c_ (*N*_d_, LWP), which allows for the relationship to be expanded by chain-rule as

[3] $$\alpha_{c}={\left.\frac{\partial \alpha_{c}}{\partial N_{d}}\right|}_{\mathrm{LWP}}\Delta N_{d}+\frac{\partial \alpha_{c}}{\partial \mathrm{LWP}}\Delta \mathrm{LWP},$$

This work focuses on the short-term effects of aerosols on cloud properties, so long-term adjustments, such as to LWP, are not included. Specifying the dependencies of *N*_d_=*N*_d_(*N*_acc_, *S*), *S*=*S*(*N*_acc,_ *w*), and *w*=*w*(*N*_acc_), the Twomey effect can be decomposed into the activation response

[4] $${\left.\frac{\partial \alpha_{c}}{\partial \ln N_{d}}\frac{\mathrm{dln}N_{d}}{\mathrm{dln}N_{\mathrm{acc}}}\right|}_{\mathrm{LWP}},$$

the cloud supersaturation “competition” response,

[5] $${\left.\frac{\partial \alpha_{c}}{\partial \ln N_{d}}\frac{\partial \ln N_{d}}{\partial S}\frac{dS}{\mathrm{dln}N_{\mathrm{acc}}}\right|}_{\mathrm{LWP}},$$

and a dynamical response (68),

[6] $${\left.\frac{\partial \alpha_{c}}{\partial \ln N_{d}}\frac{\partial \ln N_{d}}{\partial S}\frac{\partial S}{\partial w}\frac{dw}{\mathrm{dln}N_{\mathrm{acc}}}\right|}_{\mathrm{LWP}}.$$

Estimates for each term were obtained from multiple linear regression of the parameters and all partial derivative terms above were calculated holding other variables constant. Multilinear regressions were statistically significant at 95% confidence and had Pearson correlation coefficients > 0.25 (*SI Appendix,* Fig. S8–S13). The scene-based (CCN and Droplet Number) and parcel-based (Size-Resolving Lagrangian method with Doppler lidar) estimates had good correlation to both aerosol and updraft and provided enough supersaturation range to quantify the variability between aerosol conditions (*SI Appendix*, Fig. S9–S11), making them sufficient to decompose the Twomey effect into activation, competition, and dynamical responses. History-based (Hoppel minima) estimates reflect >12 -hour averages of cloud processing that average out the updraft variability, which obscures the updraft dependence of the supersaturation (*SI Appendix*, Fig. S8). The Quasi-Steady State approximation distributions show a lack of dynamic range in supersaturation (*SI Appendix*, Fig. S5 and S13) that are poor at capturing the aerosol dependence. MODIS N_d_ retrievals paired with the surface-based aerosol and updraft measurements were also used to fit multiple linear regressions (*SI Appendix*, Fig. S12), but due to the long averaging times required and the low and narrow distributions of supersaturation (*SI Appendix*, Fig. S5), they could not provide reasonable constraints on the competition response. For these reasons, only the Size-Resolving Lagrangian and CCN and Droplet Number methods were used.

## Supplementary Material

Appendix 01 (PDF)

## Data Availability

Code and data in digital archive/repository data have been deposited in Measurements and Analysis Products from Baselining the Indirect Effect by Improving Quantification of Sea Spray and Marine Sources at Ascension Island during LASIC (https://doi.org/10.6075/J0HH6K9M) (69).
