# Supplementary material for: Competition response of cloud supersaturation explains diminished Twomey effect for smoky aerosol in the tropical Atlantic
Source: Proc Natl Acad Sci U S A. 2025 Mar 24;122(13):e2412247122. doi: 10.1073/pnas.2412247122 (PMC12002179; doi:10.1073/pnas.2412247122)
Supplement: Supplementary file 1 — Appendix 01 (PDF) [file pnas.2412247122.sapp.pdf]

Supporting Information for

Competition Response of Cloud Supersaturation Explains Diminished Twomey Effect for Smoky Aerosol in the Tropical Atlantic

Jeramy L. Dedrick, Christian N. Pelayo, Lynn M. Russell*, Dan Lubin, Johannes Mülmenstädt, Mark Miller

*To whom correspondence should be addressed. Email: lmrussell@ucsd.edu.

This PDF file includes:

Supporting text S1 to S5
Figures S1 to S12
Tables S1 to S4
SI References

Supporting Information Text

S1. Cloud and Aerosol Observations

Aerosol and cloud measurements from LASIC were averaged to a common time of 2 h. Each 2 h period was classified as "clean" or "smoky" marine using metrics of aerosol number concentration and tracers of continental and combustion sources (1). The strongest smoke signal from biomass burning at Ascension Island was during the months of Jun-Oct (1, 2), while Nov-May typically had limited influence of smoke transport and were only classified as "clean". Nov-May non-clean periods were likely from local pollution sources (1, 3, 4) or dust (5) and were not used. Changes in aerosol conditions during Nov-May are interpreted as the natural variability of the baseline aerosol state, while episodic smoke intrusion during Jun-Oct allowed for contrasting clean background and smoky conditions, providing intermittent examples of anthropogenic perturbation.

Three lognormal modes characterizing Aitken (~10-80 nm), accumulation (~80-400 nm), and sea spray (>400 nm) aerosol were retrieved using an automated fitting algorithm applied to submicron size distributions measured by a TSI scanning mobility particle sizer (10-460 nm) coupled to a TSI model 3772 condensation particle counter (CPC) (6), and scattering-constrained supermicron particle contributions from a DMT Ultra-High Sensitivity Aerosol Spectrometer (60-1000 nm) (7), and a 3-wavelength integrating nephelometer (8, 9). A second 3772 CPC measured the aerosol concentration of particles larger than 10 nm (CN_{10}). The number concentration of cloud condensation nuclei (CCN) at supersaturations of 0.1-1.0% was measured using a DMT CCN counter (model 200) (10). An Aerosol Chemical Speciation Monitor (ACSM) (Aerodyne Research Inc., Billerica, MA) was used to characterize the submicron composition. The uncertainties of the surface-based aerosol measurements used in this work have been reported in Dedrick et al. (1) and references therein. Column-integrated aerosol optical depth ($\tau_{a,AERONET}$) at 340 nm were obtained from an AERONET site on Ascension Island (7.976°S, 14.415°W) (11).

The cloud optical depth (τ_{cloud}), liquid water path (LWP), layer-averaged droplet number concentration (N_d), and effective radius (r_e) were retrieved with surface-based remote sensing observations from a Multifilter Rotating Shadowband Radiometer (MFRSR) (Campbell Scientific, Logan, UT, USA) and Microwave Radiometer (MWR) (Radiometric Corporation, Boulder, CO, USA). These observations were integrated into two ARM value-added products (VAPs): MFRSRCLDOD (12) for r_e and τ_{cloud} following Min and Harrison (13) and NDROP (14) for N_d and LWP following McComiskey et al. (15). From the 4166 identified clean (2043) and smoky (2123) 2 h periods, microphysical retrievals were only evaluated for daytime overcast (>0.9 cloud fraction), low (<3 km cloud top height), single-layer, warm (>0°C) cloud scenes with optical depth >7 resulting in 1874 2 h periods. MFRSRCLDOD assumed an r_e value of 8 µm when LWP measurements were not available so these times were removed before averaging to 2 h. Zhang et al. (16) reported an uncertainty of 0.5-2.5 for MFRSRCLDOD τ_{cloud}, and found N_d and r_e retrieved from the ARM VAPs showed similar seasonal trends to aircraft, lidar, and radar retrievals in overcast stratiform cloud conditions over the eastern North Atlantic but were often biased low for r_e and high for N_d compared to the lidar and radar retrievals. The LWP uncertainty was 20 g m^{-2} when <200 g m^{-2} and up to 10% when LWP >200 g m^{-2} (14), so LWP retrievals less than 20 g m^{-2} were excluded from the analyses. LWP measurements >300 g m^{-2} were also excluded as those observations likely represented periods of heavily precipitating cumulus or stratocumulus clouds (17, 18). The boundary layer was determined to be well-mixed by calculating the difference between the lifted condensation level estimated from radiosondes and the cloud base height from the ceilometer, where a difference of <150 m represents a coupled boundary layer (19). Well-mixed, coupled boundary layers occurred for 41% (n=1707) of the combined 2 h clean and smoky periods. These criteria of 20-300 g m^{-2} LWP single-layer, daytime overcast coupled clouds resulted in 1553 2 h periods, which was 37% of the combined clean and smoky periods.

Cloud base vertical velocity, *w*, was retrieved from a surface-based Doppler lidar (DL; Halo Photonics, Worcestershire, UK) and a Ka-band ARM Zenith Radar (KAZR) (20). *w* from the DL was estimated by first identifying the cloud base using a ceilometer (Model CL31, Vaisala, Vantaa, FIN) and then determining the DL vertical velocity at that height gate over a 30 min time window (21). Here we use the median value from this time window, of which only positive values are retained as

an estimate of *w*. *w* from KAZR was retrieved using reflectivity (Z_R) and Doppler velocity (V_d) measurements obtained from Active Remote Sensing of Clouds (ARSCL) value-added product (VAP) (22). Below-cloud drizzle/virga were identified in the Z_R profiles following the approach of Wu et al. (23). Once these precipitating hydrometeors were removed from V_d profiles, V_d at the height of cloud base (identified by the ceilometer or Doppler lidar) represented the vertical air motion and hence the updraft (24), w_{KAZR}. Many of the LASIC clouds were identified as having drizzle at or below cloud base using the Wu et al. *(22*) approach, meaning that w_{KAZR} was retrieved for only 10% of the available 2 h measurements.

Additional atmospheric state measurements were obtained from value-added products (VAP), such as the interpolated radiosonde (INTERPSONDE) (25), and from the Modern-Era Retrospective Analysis for Research and Applications, Version 2 (MERRA-2) (26). INTERPSONDE was based on two and four-times daily radiosondes deployed at Wideawake Airfield (80 m ASL), located 4 km away from the aerosol measurement site on Green Mountain, and provided measurements of temperature, relative humidity, and pressure throughout the atmospheric column that were re-gridded to 1-min time resolution and 332 height levels from the surface-based to approximately 40 km. We used geopotential height and wind speed at the 700 hPa pressure level from MERRA-2 at a temporal resolution of 6 h for the years of 2010-2020.

A total sky imager (Yankee Environmental Systems, Inc., Turner Falls, MA) provided the fractional sky cover of clouds that were distinguished as “opaque” or “thin” based on hemispheric sky images. The opaque and thin fractions were combined to give a total cloud fraction for each 2 h. Cloud albedo was calculated from clear-sky and all-sky downwelling broadband shortwave irradiances from surface-based pyranometer measurements using RADFLUXANAL (27).

Aerosol and cloud properties were retrieved from 1 km resolution MODIS Collection 6 Version 6.1 Level-2 cloud product and 10 km resolution MODIS Collection 6 Version 6.1 Level-2 Dark Target and Deep Blue combined Aerosol product from both the Terra (MOD04 and MOD06) and Aqua (MYD04 and MYD06) satellites. A 1° x 1° bounding box surrounding Ascension Island (-7.9670°, -14.3498°) was selected for this analysis. For quality screening, MODIS observations were only retained for overcast (>0.9 cloud fraction), low-level (<3 km cloud top height), single-layer cloud scenes (28). Cloud effective radius and cloud optical depth retrievals were only included when individual uncertainty was less than 50% of the measured value to minimize retrieval uncertainty (29). Cloud droplet number concentrations were computed using the cloud optical depth, effective radius, and cloud top temperature and pressure (30).

Satellite and surface-based measurements have shown that multi-level cloud systems consisting of cumulus and stratocumulus clouds are prevalent in this trade-wind sector of the South Atlantic, particularly during smoky conditions (Jun-Oct) (up to 70%) (31, 32). The average single-layer, low-level cloud base heights estimated by the ceilometer were 427±182 m (mean±standard deviation) during Nov-May with an average geometric thickness of 582±341 m. Similar results were found for clean conditions during Jun-Oct (508±236 m base height, 618±296 m geometric thickness). For smoky conditions, the mean cloud base height and thickness were 549±214 m and 543±297 m, respectively.

Clean conditions were predominantly characterized by low condensation nuclei number concentrations >10 nm (CN_{10}; an effective proxy for the “total” number of aerosol particles), low accumulation-mode aerosol number concentration (N_{acc}; the number of particles contributing the greatest fraction of CCN), and low $\tau_{a,AERONET}$ (Fig. S1a-c). The number concentration of cloud droplets (N_d) was similarly low with an average of 106±87 cm^{-3} for clean conditions (Fig. S1e) and was consistent with low (<500 cm^{-3}) marine stratus and cumulus N_d measured over the open ocean from the surface (15, 33-35), in-cloud (36, 37), and from satellites (38) (Table S2). Aerosol and droplet concentrations doubled during smoky conditions, with mean N_d of 213±195 cm^{-3} (Fig. S1a-c,e). The mean smoky N_d was on the lower-end of N_d from smoky and polluted marine cloud measurements previously reported in the South Atlantic (300-500 cm^{-3}) (37) and the mean value of 611±191 cm^{-3} in “mixed” smoky clouds over Ascension Island reported by de Graaf et al. (33). The difference in LASIC measurements between the clean and smoky conditions were statistically significant at 95% confidence using a two-tailed t test, indicating a distinct seasonal change in the

number of droplets when smoky aerosol increased the number concentration of particles in the Ascension Island marine boundary layer.

Smoky marine conditions had smaller cloud droplets, with a 3 μm decrease in mean r_e between clean (14±4 μm) and smoky (11±4 μm) conditions (Fig. S1f). The change in the statistical distribution of r_e between the two conditions was statistically significant at 95% confidence. Similar clean marine r_e measurements of 5-30 μm have been reported in the Northeast Atlantic (35, 39), Northeast Pacific (15, 36), and the Arctic (40) (Table S2). Comparable reductions in mean r_e (5-10 μm) have been observed in polluted marine clouds from surface-based and in-cloud measurements (33, 36, 41) and satellite retrievals (42) (Table S2). The mean clean and smoky r_e retrieved using LASIC measurements was approximately 10 μm larger than Ascension Island values retrieved by de Graaf et al. (33). This difference is likely attributable to the LASIC retrieval of r_e being a cloud layer mean, while de Graaf et al. (33) retrievals were evaluated at cloud-base, where a lower liquid water content may have contributed to smaller droplet sizes in non-precipitating adiabatic clouds (43).

In contrast to the clean and smoky differences in aerosol particle and cloud droplet distributions, the cloud macrophysics, thermodynamic, and turbulence properties showed no clear distinction between clean and smoky conditions (Fig. S1d,g,h). Clean condition LWP was only 10 g m^{-2} higher than smoky (Fig. S1g), consistent with previously observed mean differences in smoky and "less smoky" conditions at Ascension Island (31). The average LWP for clean and smoky conditions (128±71 g m^{-2}) was in a similar range compared to stratus clouds observed in the Northeast Pacific (~125 g m^{-2}) (15), precipitating stratus in the Northeast Atlantic (~150 g m^{-2}) (44), and shallow trade wind cumulus clouds (50-100 g m^{-2}) (17, 45). Boundary layer stability, diagnosed from a calculation of the lower tropospheric stability (LTS) (46), showed no difference between clean and smoky conditions as the means and standard deviation for clean and smoky were identical and both were predominantly stable (LTS of 15-17) (47, 48). Retrievals of cloud base Doppler lidar *w* (Fig. S1d) also showed no difference between clean and smoky conditions with identical means in clean and smoky marine conditions and were consistent with previous measurements of marine stratocumulus (39, 49, 50) and fair weather trade wind cumulus (51, 52).

S2. Aerosol-Cloud Interaction Calculations

LWP has been separated by low (optically thinner) and high (optically thicker) values around the LASIC median value of 115 g m^{-2}, after the exclusion of LWP values >300 g m^{-2} (Text S1, Fig. S1g). This separation approximately distinguishes precipitating (115-300 g m^{-2}) and non-precipitating (20-115 g m^{-2}) clouds (34, 44, 53). ACI were evaluated for single-layer clouds. Differences in the South Atlantic basin synoptic regimes did not affect the surface meteorology or cloudiness and did not show clear differences in the microphysical responses to aerosol at Ascension Island based on k-means clustering of the large-scale circulation (Text S4, Table S3, Fig. S9), so were not used as controlling factors.

The LASIC r_e response to surface-based aerosol measurements (Fig. S2) is more sensitive than some reported satellite aerosol and microphysical retrievals in clean and polluted marine conditions that fall within the range of -0.08 to 0.18 (42, 54, 55) (Table S2). LASIC N_{acc} ACI_N for clean and smoky marine conditions (0.67-0.68; Fig. S2, Table S2) was directly comparable to global (0.62-0.66) (56, 57) and South Atlantic (~0.25-0.6) ranges from satellite estimates (56) (Table S2).

For clean and smoky marine conditions, optically thinner clouds had a stronger microphysical response to the aerosol changes than thick clouds, consistent with weaker entrainment-mixing, stronger adiabaticity, and cloud base droplet evaporation that result in greater microphysical sensitivity to aerosol and higher ACI values in thin, stable low clouds (48, 58). Weaker ACI_r in thick clouds was likely associated with increased collision-coalescence in thick, drizzling clouds (r_e>14 μm) (18) that buffered aerosol effects on the microphysical properties (15, 59). Thick, clean and smoky clouds during LASIC were 10-30% more likely to have r_e>14 μm than thin clouds, suggesting a reduced aerosol effect in thick clouds. High *w* (w>0.45 m s^{-1}) also appeared to be associated with slight increases in the strength of ACIr for N_{acc} in smoky clouds (Table S4). This is consistent with stronger updrafts increasing cloud supersaturation and broadening the droplet spectrum with increases in aerosol concentration (35, 60) and a potential *w*

limitation on droplet activation. The lack of correlation in smoky conditions using MODIS τ_a and surface-based aerosol properties highlight the complexity of the aerosol environment in these conditions, where $r_{e,MODIS}$ would be influenced by aerosol above, within, and below clouds that is not fully resolved at cloud edges (55, 61).

S3. History, Scene, and Parcel-based Supersaturation Calculations and Uncertainty

Four approaches to estimate cloud supersaturation were evaluated using the surface-based aerosol measurements and retrievals of microphysical properties and updraft. The first two methods use history-based approaches to constrain the supersaturation based on the cumulative relationship between the unactivated and activated aerosol distribution; the third approach uses a "scene-based" approach where microphysical properties of the cloud scene are used to constrain supersaturation; the last method used parcel-based approaches to evaluate supersaturation based on the local updraft velocities retrieved.

(1) Hoppel Minimum and Hygroscopicity method used the κ-Kohler parameterization (62) to relate the Hoppel minimum diameter, D_{HM}, as the critical diameter, with the accumulation-mode hygroscopicity (κ_{acc}) calculated from ACSM measurements of submicron non-refractory composition. In the remote marine boundary layer, D_{HM} represents the average minimum size at which aerosol formed cloud droplets that grew by cycling in multiple non-precipitating clouds, with the retrieved value reflecting the lowest critical diameter of the clouds that processed the aerosol. This metric was shown to be evidence of an aerosol feedback on cloud supersaturation because it correlated moderately to the accumulation-mode concentration of clean marine aerosol size distributions during LASIC (1).

(2) Hoppel Minimum and CCN method used the number of particles above the Hoppel minimum diameter (N_{HM}) calculated from the CCN supersaturation spectra as the supersaturation at which N_{HM}=$N_{CCN,\%}$, following Gong et al. (63) but adapted to surface-based observations, where N_{HM} was found by integrating the aerosol number size distribution above D_{HM}. The adaptation to surface-based measurements means this approach also reflects the minimum critical diameters from the ensemble of cloud supersaturations that processed the aerosol.

(3) CCN and Droplet Number method used N_d calculated from the CCN supersaturation spectra as the supersaturation at which N_{CCN}=N_d (63-65). N_d is retrieved radiometrically from MWR and MFRSR or MODIS, and thus represents an ensemble of cloud scenes that may include a variety of updraft velocities.

(4) Size-Resolving Lagrangian methods were calculated for single (66) and multi-modal (67) aerosol representations , a parcel-based parametrization commonly used in global climate models (GCM) to predict droplet activation (68-70), using radiosondes, submicron hygroscopicity, the measured *w* (from the Doppler lidar and KAZR), and the fitted Aitken, accumulation, and sea spray modes from the measured aerosol size distributions (1). The single mode representation was computed assuming only accumulation-mode aerosol.

The Abdul-Razzak et al. (66) (S_{AR98}) parameterization for a single-mode aerosol follows as:

$$S_{AR98} = \frac{S_m}{\left[f_1(\ln\sigma)\left(\frac{\zeta}{\eta}\right)^{3/2} + f_2(\ln\sigma)\left(\frac{{S_m}^2}{\eta}\right)^{3/4} \right]^{1/2}} \qquad \text{(S2).}$$

The Abdul-Razzak and Ghan (67) (S_{AG00}) parameterization for multi-mode aerosol follows as:

$$S_{AG00} = 1/\left\{\sum_{i=1}^{I} \frac{1}{S_{mi}^2}\left[f_i\left(\frac{\zeta}{\eta_i}\right)^{\frac{3}{2}} + g_i\left(\frac{S_{mi}^2}{\eta_i + 3\zeta}\right)^{\frac{3}{4}}\right]\right\}^{1/2} \quad \text{(S3).}$$

S_m represents the critical supersaturation from the (internally-mixed) submicron hygroscopicity and Kohler theory. The functions f_i and g_i depend on the modal width from the fitted aerosol size distributions, while ζ and η account for the updraft velocity and thermodynamic constants, respectively. Subscript *i* represents each of the modes considered. S_{AG00} variability may also explain microphysical changes in the cloud properties over Ascension Island as represented by weak correlations (r=0.2-0.4) with N_d and r_e in clean and smoky conditions (Fig. S5). These results show increases in cloud droplet radii with S_{AG00} and higher S_{AG00} (>0.2%) in low N_d (N_d<200 cm^{-3}) conditions.

A quasi-steady approximation is included for comparison. The Quasi-Steady State method was calculated from the empirical parameterization assuming a quasi-steady state balance on a parcel model between the increase of saturation by cooling in the updraft and depletion of vapor by the condensation of activated particles (71), constrained by the retrieved *w* and N_d, and the temperature and pressure measured at cloud base from radiosondes (Text S1). Because the mean droplet radius ($\bar{r}_d$) was not directly retrieved, we have approximated the value assuming the droplet distribution can be represented by a modified gamma distribution constrained by the retrieved r_e, N_d, and assuming an effective variance of 0.2 for stratiform clouds (72). This parcel-based approach reflects the conditions of a single updraft after reaching steady state, and so is included for comparison even though it is not equivalent to the other methods. Quasi-steady state supersaturation was calculated following Lamb and Verlinde (71):

$$S_{qs} = \frac{Aw}{\bar{r}_d N_d} \quad \text{(S1),}$$

where *A* is a parameter that considers radiosonde-retrieved cloud base temperature, pressure, and thermodynamic constants (73). $\bar{r}_d$ (the mean radius), N_d was the droplet number concentration, both from the surface-based radiometric retrievals, and *w* is the cloud base updraft velocity from the Doppler lidar and KAZR. The parcel-based supersaturation using "quasi-steady state" assumptions (73) has notably smaller values than the ensemble-based retrievals (Fig. 3, Fig. S4), likely because aerosol composition or CCN activity were not included as variables and because a longer adjustment time was needed for quasi-steady state.

The highest correlations between supersaturations were for the two pairs of similar (non-independent methods) (Fig. S4), namely the two history-based methods (Hoppel minimum and hygroscopicity and Hoppel minimum with CCN Number) and the two parcel-based methods (Size-Resolving Lagrangian and the Quasi-Steady State approximation). The next strongest correlations were observed for Size-Resolving Lagrangian and the cloud scene-based method (CCN and Droplet Number), which also showed similar statistical distributions (Fig. 3). The intercomparison between these two independent supersaturation retrievals along with their dynamic range of updraft (Methods) make them suitable to calculate aerosol effects on clouds.

The uncertainty range in the 5 supersaturation retrievals were estimated using a Monte Carlo approach based on the measurement uncertainties (74, 75). Hypothetical supersaturations were calculated assuming uncertainty in the aerosol size distribution number (10%) and diameter (2.5%), cloud base temperature (0.3°C) and pressure (0.5 hPa) from radiosondes, measured CCN number concentration (4%) and instrument supersaturation (3%), measured non-refractory mass fraction (30%), measured updraft variability (standard deviation of fitted probability distribution function), and the propagated errors of the microphysical retrievals (N_d, r_e) for each 2 h period which are dependent upon the LWP, mixing parameter, optical depth, adiabatic condensation rate, and spectral width (12, 14). In each calculation of supersaturation, the variables were randomly sampled 1000 times from a normal distribution with the mean as the measured value and standard deviation as the uncertainty. A geometric mean and standard deviation of the 1000 samples were calculated for each 2 period to derive upper and lower bounds of the uncertainties. Based on this Monte Carlo approach, we find the following uncertainties in supersaturation for each method:

(1) Hoppel Minimum and Hygroscopicity: ±18%
(2) Hoppel Minimum and CCN: ±22%
(3) CCN and Droplet Number: ±28%
(4) Size-Resolving Lagrangian: ±18% for multimode (21% for single mode)

The higher uncertainties associated with the supersaturation methods using the droplet number are likely due to the uncertainty in the interpolation (Hoppel Minimum and CCN, CCN and Droplet Number). The Quasi-Steady State approximation had a larger uncertainty at ±33% than the four methods used here, which was a result of the propagated errors of the microphysical retrievals (Quasi-Steady State) that can be up to 13%.

S4. Synoptic Regime Classification using k-means Clustering

k-means clustering was performed on the MERRA-2 reanalysis dataset to identify the main South Atlantic synoptic regimes and their potential influences on surface meteorology and the boundary layer cloud structure at Ascension Island. Here, the daily 700 hPa geopotential height anomaly (daily deviation from the monthly mean) was normalized by the monthly standard deviation (daily anomaly divided by the monthly standard deviation) and used as input to the MATLAB *kmeans.m* algorithm. Means and variance were calculated from the 11-year climatology of 2010-2020. The Southeast Atlantic basin was selected covering the latitude range of -40°S to 0°S and a longitude range of 40°W to 20°E. This regional selection was consistent with Gaetani et al. (76), which aims to reduce the strong influence of South Atlantic midlatitude modes that mask variability in the tropics. The number of clusters ranged from 1 to 10 and were statistically clustered using a squared Euclidean distance metric, 1000 replications, and 1000 iterations. Four clusters were ultimately chosen as the optimal number for this work as they effectively described the regional synoptic variability (Fig. S9). Namely, the variability, as expected, was largely driven by the position and strength of the South Atlantic subtropical high-pressure system. Cluster 1 (SA+) was the anomalously strong South Atlantic high and cluster 2 (SA-) was the anomalously weak South Atlantic high. Clusters 3 and 4 are characterized by basin-wide anomalously low geopotential height (cluster 3, BA-) and anomalously high geopotential height (cluster 4, BA+). These clusters had no discernible impact on the Ascension Island surface meteorology or macroscale cloud properties (Fig. S6; Table S3). The differences between the clusters for each of the assessed variables were not statistically significant at 95% confidence using a two tailed t test. SA+ (k=1).

S5. Radiative Forcing Calculations

The radiative forcing caused by aerosol-cloud interactions, RF_{ACI}, was calculated following Quaas et al. (77) for each of the Twomey responses for combined marine conditions (clean+smoky) as:

$$RF_{ACI,act.} = -F_{sw}^{\downarrow} \cdot \left\{ \frac{\partial \alpha_c}{\partial \ln N_d} \frac{\partial \ln N_d}{\partial \ln N_{acc}} \bigg|_{\mathrm{LWP}} \right\}_{\mathrm{combined}} \cdot \Delta \ln N_{acc} \quad \text{(S3)}$$

$$RF_{ACI,comp.} = -F_{sw}^{\downarrow} \cdot \left\{ \frac{\partial \alpha_c}{\partial \ln N_d} \frac{\partial \ln N_d}{\partial S} \frac{\partial S}{\partial \ln N_{acc}} \bigg|_{\mathrm{LWP}} \right\}_{\mathrm{combined}} \cdot \Delta \ln N_{acc} \quad \text{(S4)}$$

$$RF_{ACI,inv.} = -F_{sw}^{\downarrow} \cdot \left\{ \frac{\partial \alpha_c}{\partial \ln N_d} \frac{\partial \ln N_d}{\partial S} \frac{\partial S}{\partial \mathrm{w}} \frac{\partial \mathrm{w}}{\partial \ln N_{acc}} \bigg|_{\mathrm{LWP}} \right\}_{\mathrm{combined}} \cdot \Delta \ln N_{acc} \quad \text{(S5).}$$

$F_{sw}^{\downarrow}$ is the daily mean downward solar radiative flux density computed as the means of the daily-averaged downwelling shortwave radiation retrieved from the RADFLUXANAL ARM value-added product (27, 78) averaged for the entire LASIC campaign. $\Delta \ln N_{acc}$ is the relative anthropogenic perturbation in accumulation-mode aerosol, (77, 79):

$$\Delta \ln N_{acc} = \frac{\left(\overline{N_{acc}^{\mathrm{PD}}} - \overline{N_{acc}^{\mathrm{PI}}}\right)}{\overline{N_{acc}^{\mathrm{PD}}}} \quad \text{(S6).}$$

N_{acc}^{PD} and N_{acc}^{PI} represent the present-day (smoky) and pre-industrial (clean) accumulation-mode number concentration, respectively, with overbars denoting the mean for those conditions.

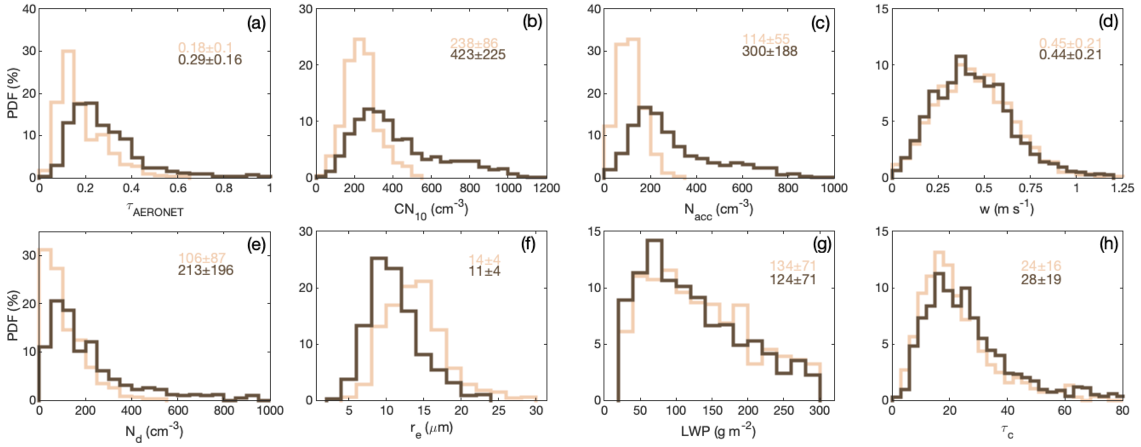

Fig. S1. Probability density functions (PDFs) of aerosol, microphysical, and updraft properties during LASIC clean (beige) and smoky (brown) marine conditions for all 2 h averaged periods. (a) aerosol optical depth retrieved from AERONET, τ_{AERONET}, (b) Condensation nuclei number concentration >10 nm (CN_{10}), (c) accumulation mode aerosol number concentration (N_{acc}), (d) median cloud base updraft velocity (*w*), (e) cloud droplet number concentration (N_d), (f), cloud droplet effective radius (r_e), (g) liquid water path (LWP), (h) cloud optical depth (τ_c).

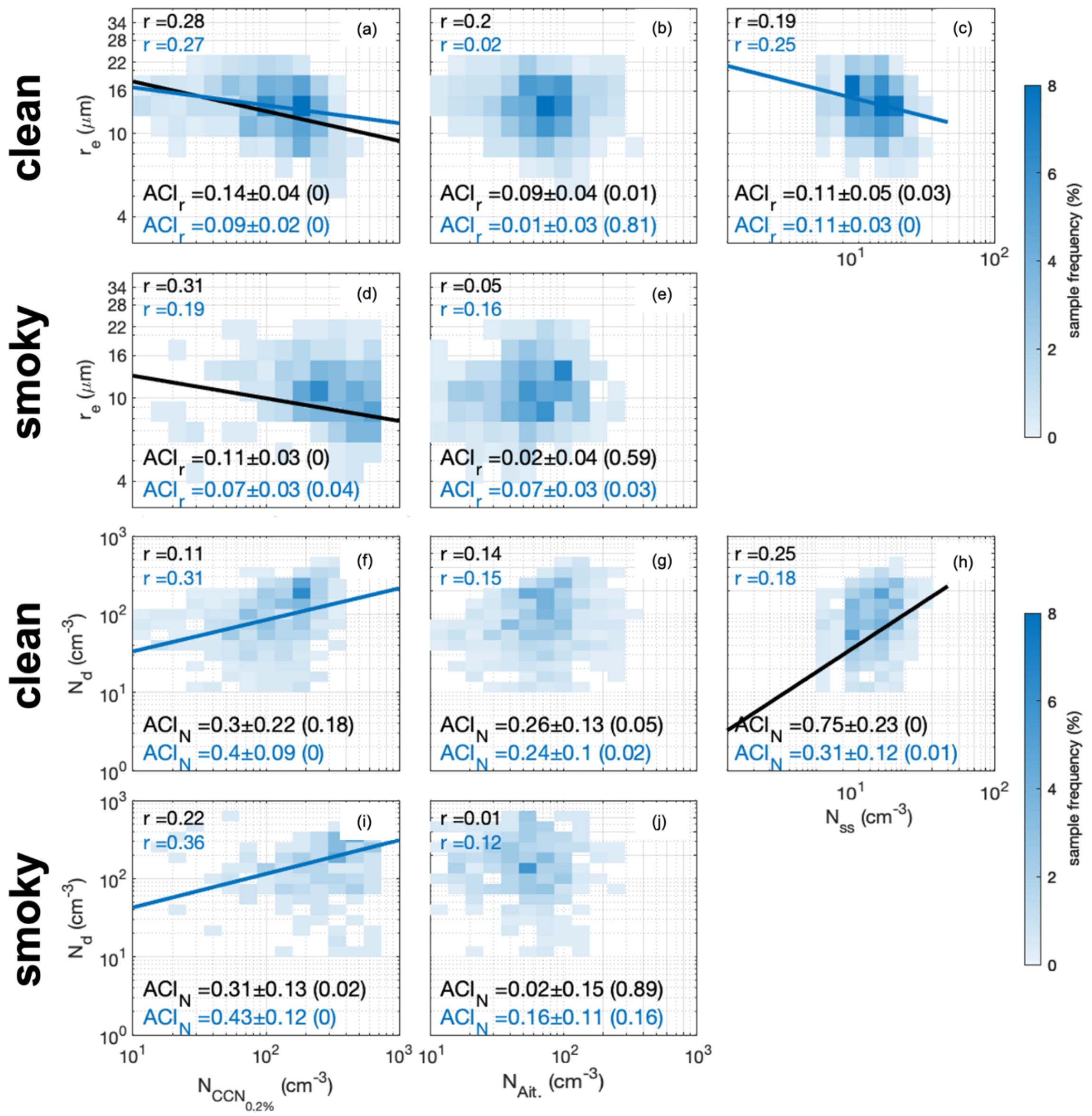

Fig. S2. Joint histograms of cloud droplet effective radius (r_e) and number concentration (N_d) versus aerosol proxies (ACI_r, ACI_N) during clean and smoky conditions. Color scale represents the percent of sample points in each bin. ACI indices at bottom of each panel computed as log-log slope and standard error (±) from linear regression with Pearson correlation coefficient (r) at top left and *p* value in parentheses. Regression fits shown in bold and as lines for statistically significant (p<0.05, two-tailed t test) fits that were at least weakly correlated (r>0.25). ACI separated by low (20-115 g m^{-2}) (black line fit) and high (115-300 g m^{-2}) (blue line fit) liquid water path. Aerosol proxies shown are the concentrations of CCN at 0.2% supersaturation ($N_{CCN0.2\%}$), Aitken-mode particles ($N_{Ait.}$), and sea spray-mode particles (N_{ss}). Note, the sea spray-mode was not retrieved during smoky conditions because of scattering constraint limitations (1, 9).

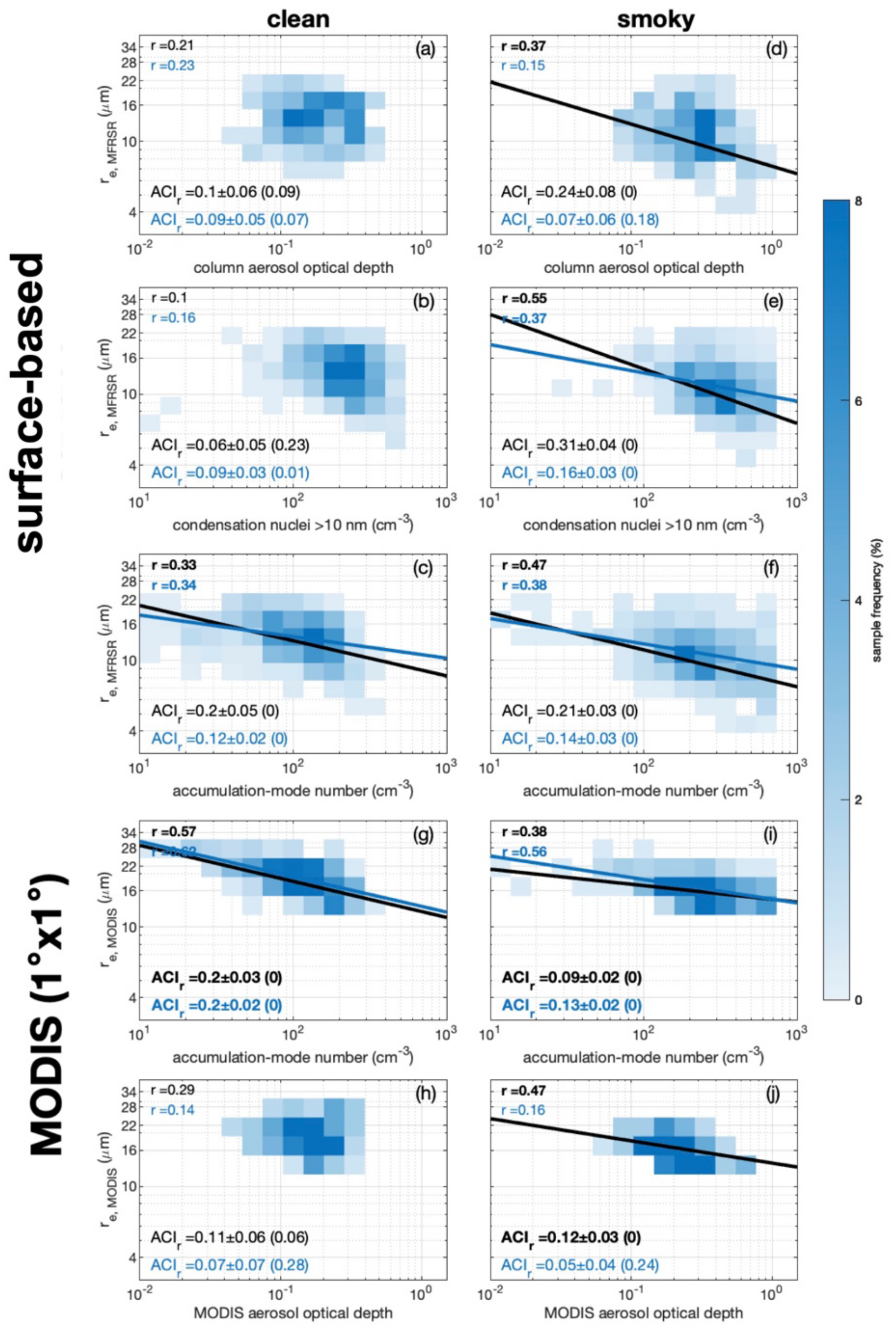

Fig. S3. Joint histograms of cloud droplet number concentration (r_e) versus aerosol proxies (ACI_r) in clean (left) and smoky (right) conditions. (A-F) Surface-based droplet number and (G-J) satellite-retrieved droplet number from the Moderate Resolution Imaging Spectroradiometer (MODIS) are compared to surface-based and satellite aerosol metrics. Color scale represents the percent of sample points in each bin. ACI_r computed as log-log slope and standard error (±) from linear regression with Pearson correlation coefficient (r) at top left and *p* value in parentheses. Regression fits shown in bold and as lines for statistically significant (p<0.05, two-tailed t test) fits that were at least weakly correlated (r>0.25). ACI_r separated by low (20-115 g m^{-2}) (black line fit) and high (115-300 g m^{-2}) (blue line fit) liquid water path.

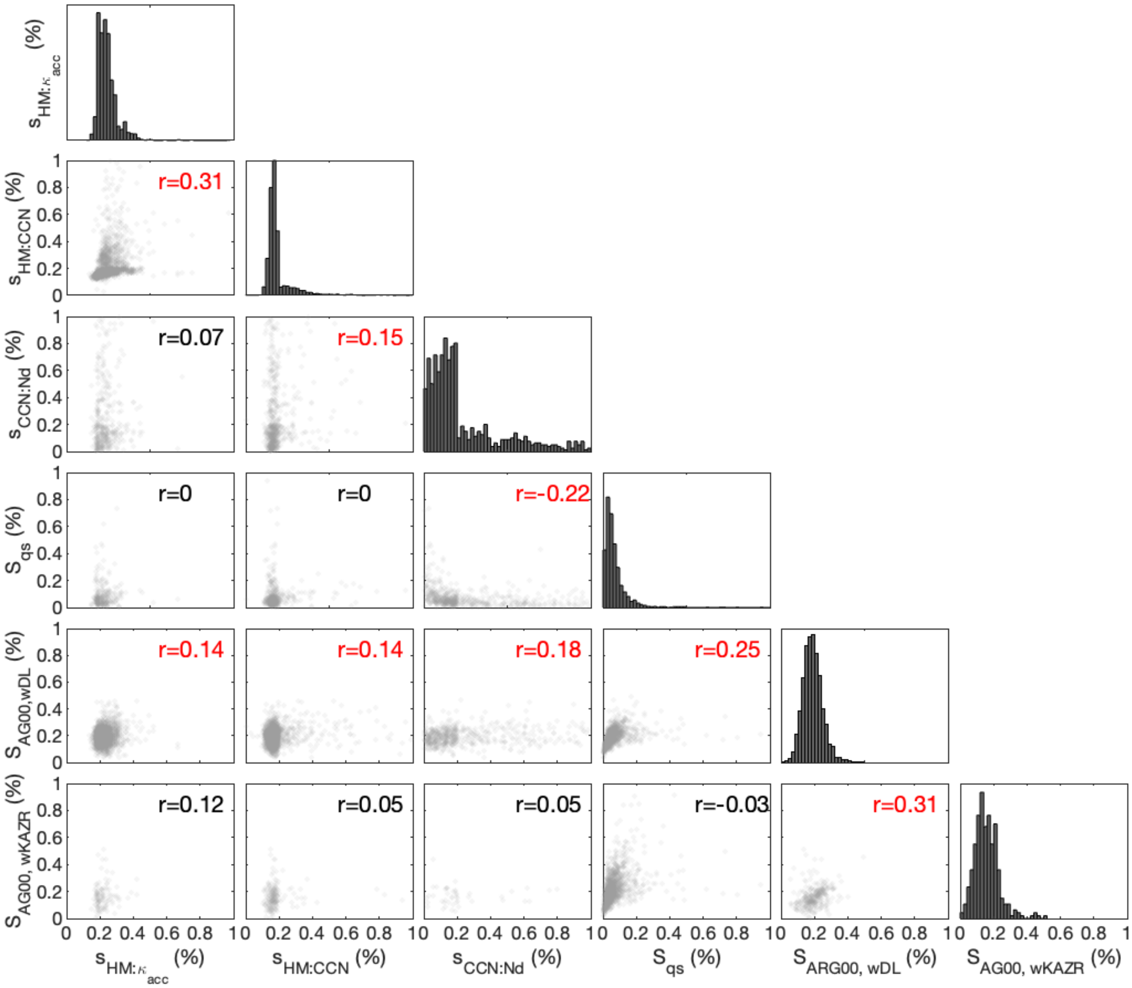

Fig. S4. Correlation matrix of effective and parameterized supersaturations. Statistically significant ($p<0.05$, two-tailed t test) Pearson correlation coefficients are colored red. Hoppel minimum and hygroscopicity ($s_{HM:\kappa acc}$), Hoppel minimum and CCN ($s_{HM:CCN}$), CCN and Droplet number ($s_{CCN:Nd}$), Quasi-steady state (S_{qs}), Size-resolving Lagrangian with Doppler lidar ($S_{AG00,wDL}$) and Ka Radar ($S_{AG00,wKAZR}$).

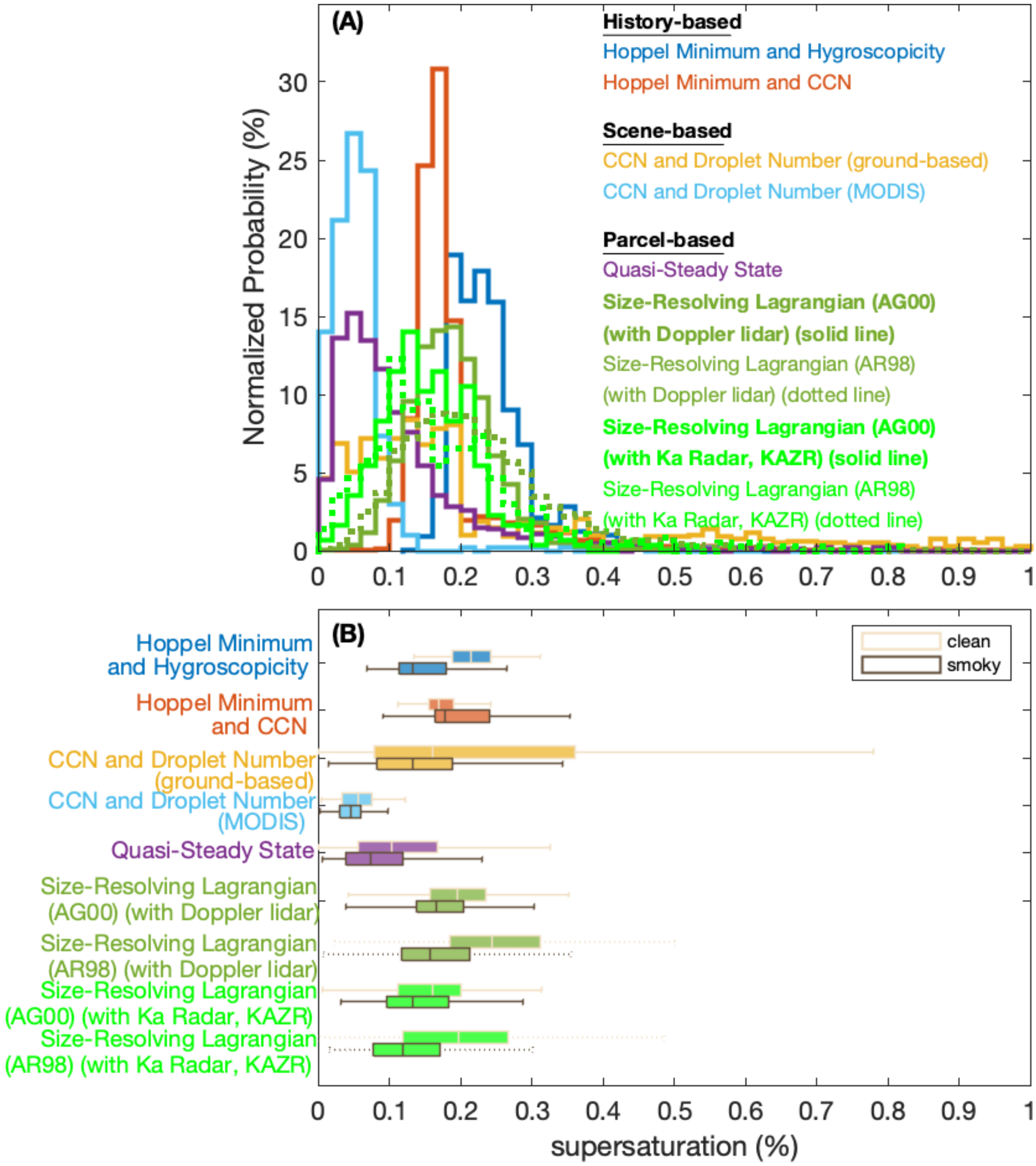

Fig. S5. Same as Fig. 3 with the inclusion of the Scene-based method using MODIS droplet number (light blue), the Quasi-Steady State method (purple), and the Size-Resolving Lagrangian method for a single, accumulation-mode (S_{AR98}) using Doppler lidar (dark green with dotted whiskers) and KAZR (light green with dotted whisker).

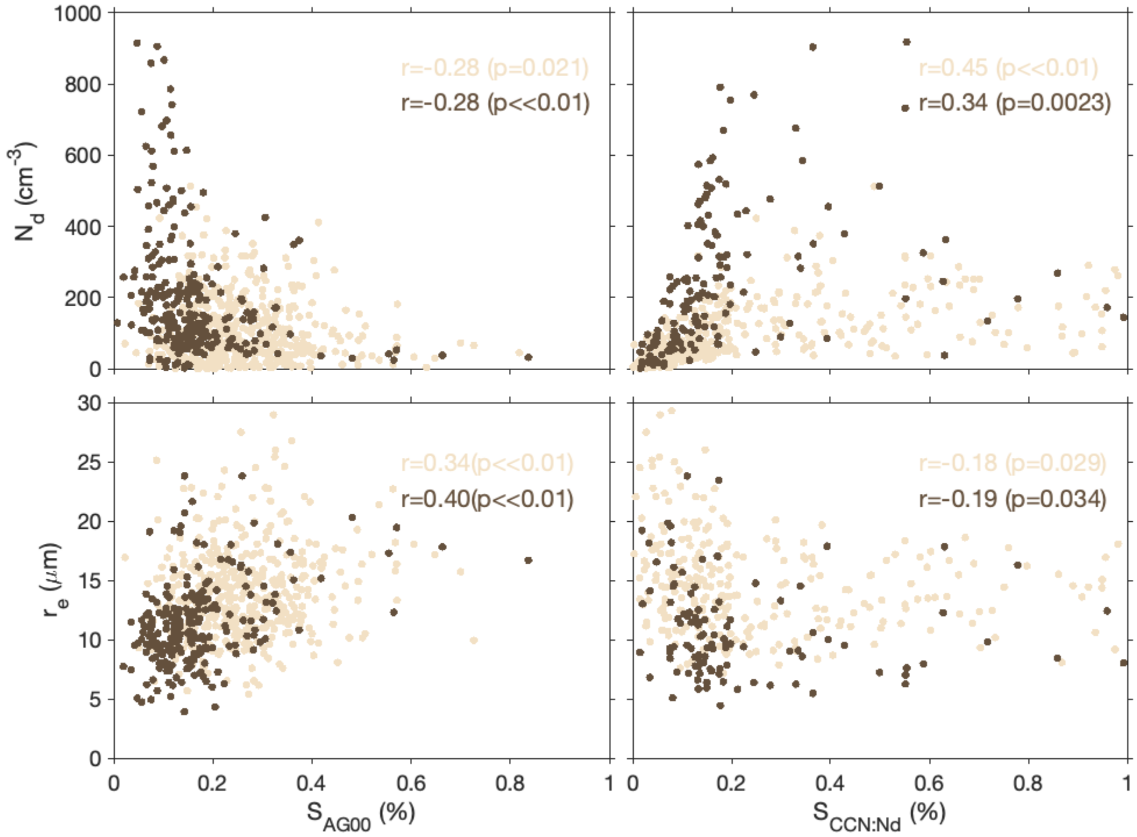

Fig. S6. Scatter plots of Multi-Mode Size-Resolving Lagrangian Method with Doppler lidar updraft (S_{AG00}, left) and CCN and Droplet number ($S_{CCN:Nd}$) supersaturations versus N_d (top) and r_e (bottom) for clean (beige) and smoky (brown) conditions. Pearson correlation coefficients between variables and their p values are shown at the upper right of each panel.

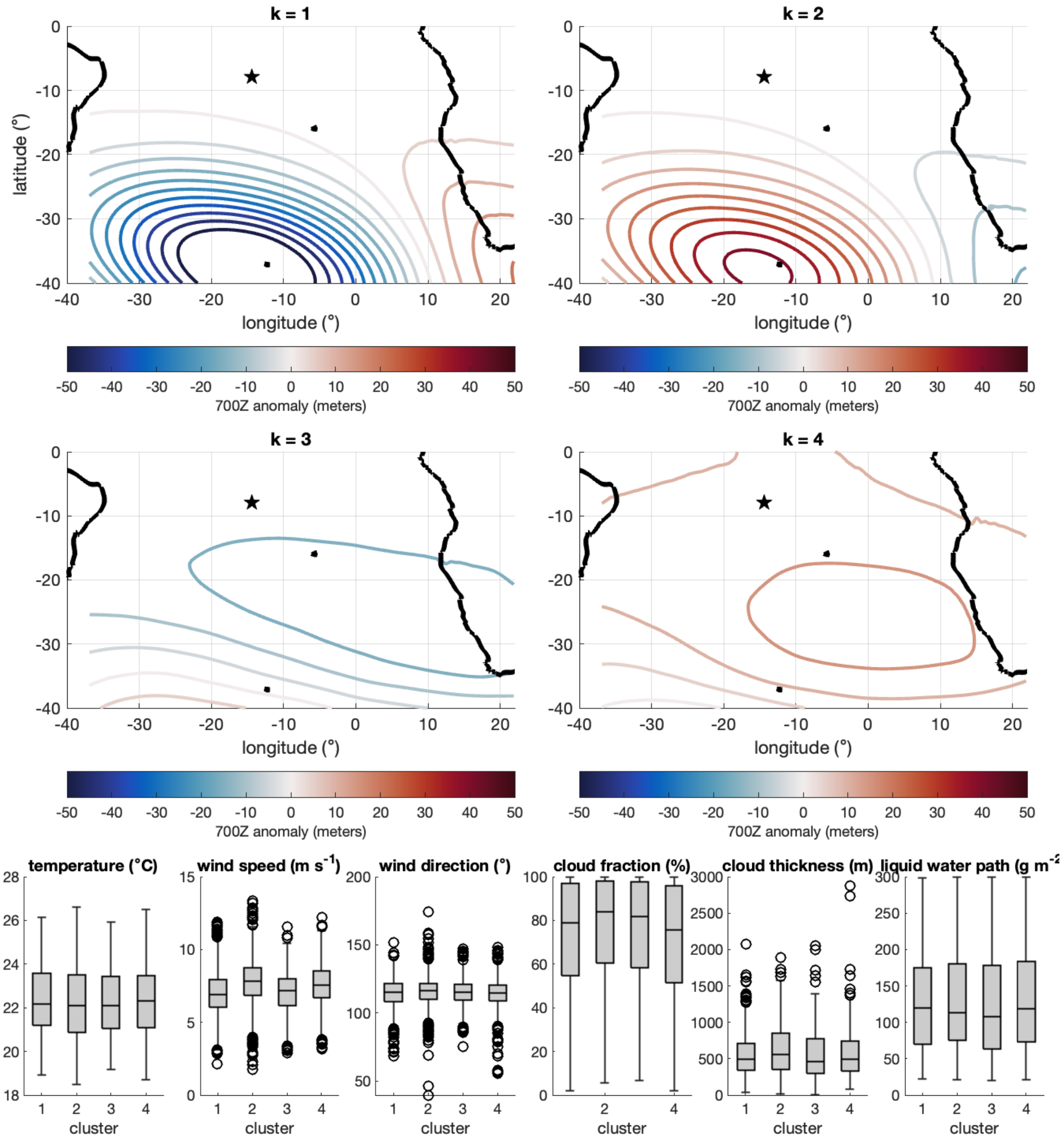

Fig. S7. Composites of synoptic regime k-means clustering. (top) Average of daily mean 700 hPa geopotential height anomaly on Southeast Atlantic map for each cluster. Location of Ascension Island identified with black star at 8°S, 14°W. (bottom) Box-and-whisker plots of selected meteorological and cloud variables for each cluster.

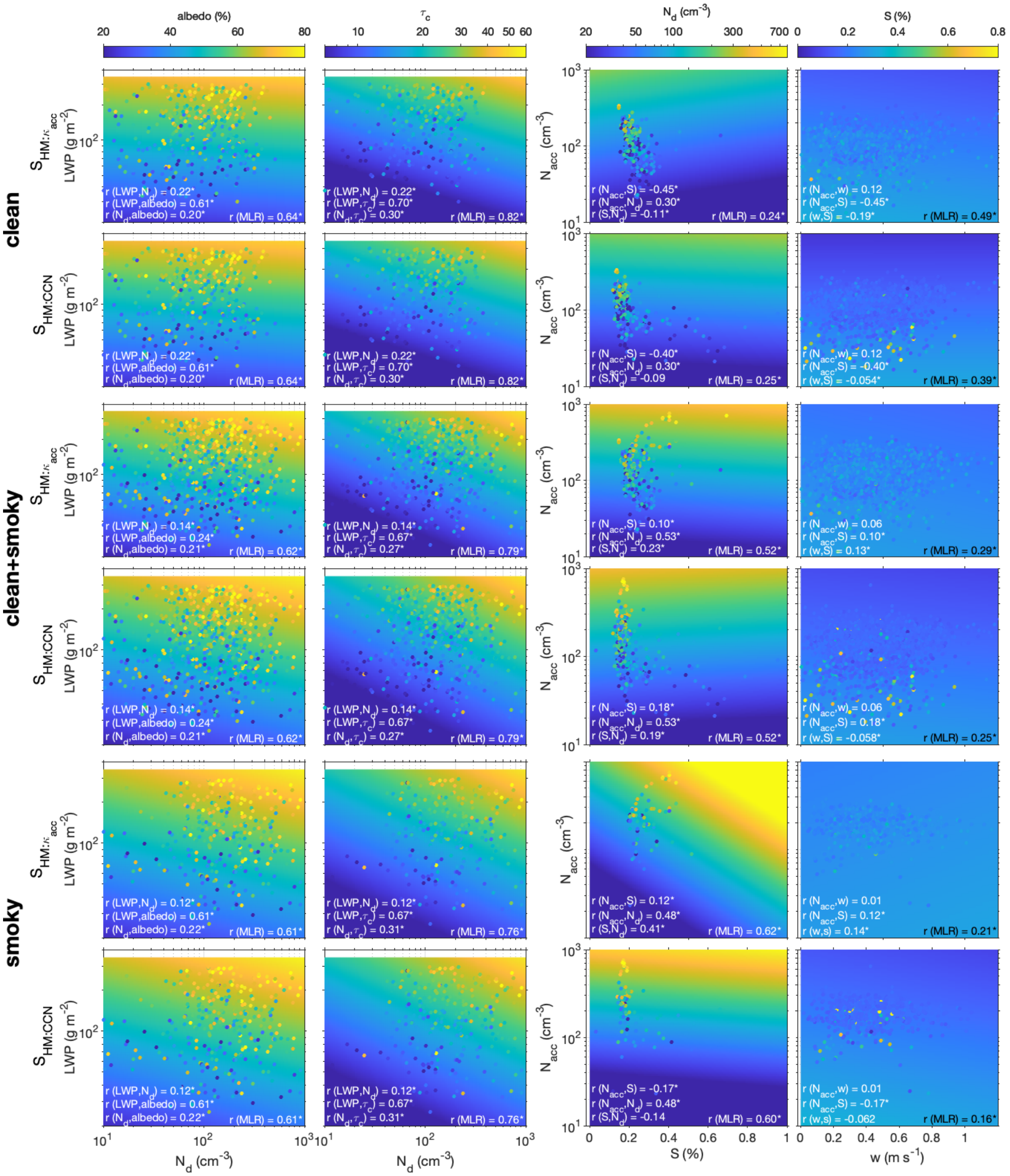

Fig. S8. Surfaces fit by multi-linear regression (contours) based on the scattered points from observations for Twomey effect decomposition using history-based supersaturations: Hoppel minimum and hygroscopicity ($S_{HM:\kappa_{acc}}$) and Hoppel minimum and CCN ($S_{HM:CCN}$). Observations are from radiometerically-retrieved measurements (albedo, N_d, LWP, τ_c) and aerosol measurements (N_{acc}, S). The LWP measurements range was restricted to 20-300 g m^{-2}. Vertically, the panels are separated by clean (top), combined (clean+smoky; middle), and smoky (bottom) conditions. Pearson correlation coefficients are shown at bottom left of each panel for singular variable pairs (in parenthesis) and at right for the multi-linear regression (MLR). Statistically significant ($p<0.05$) correlations are represented with an asterisk.

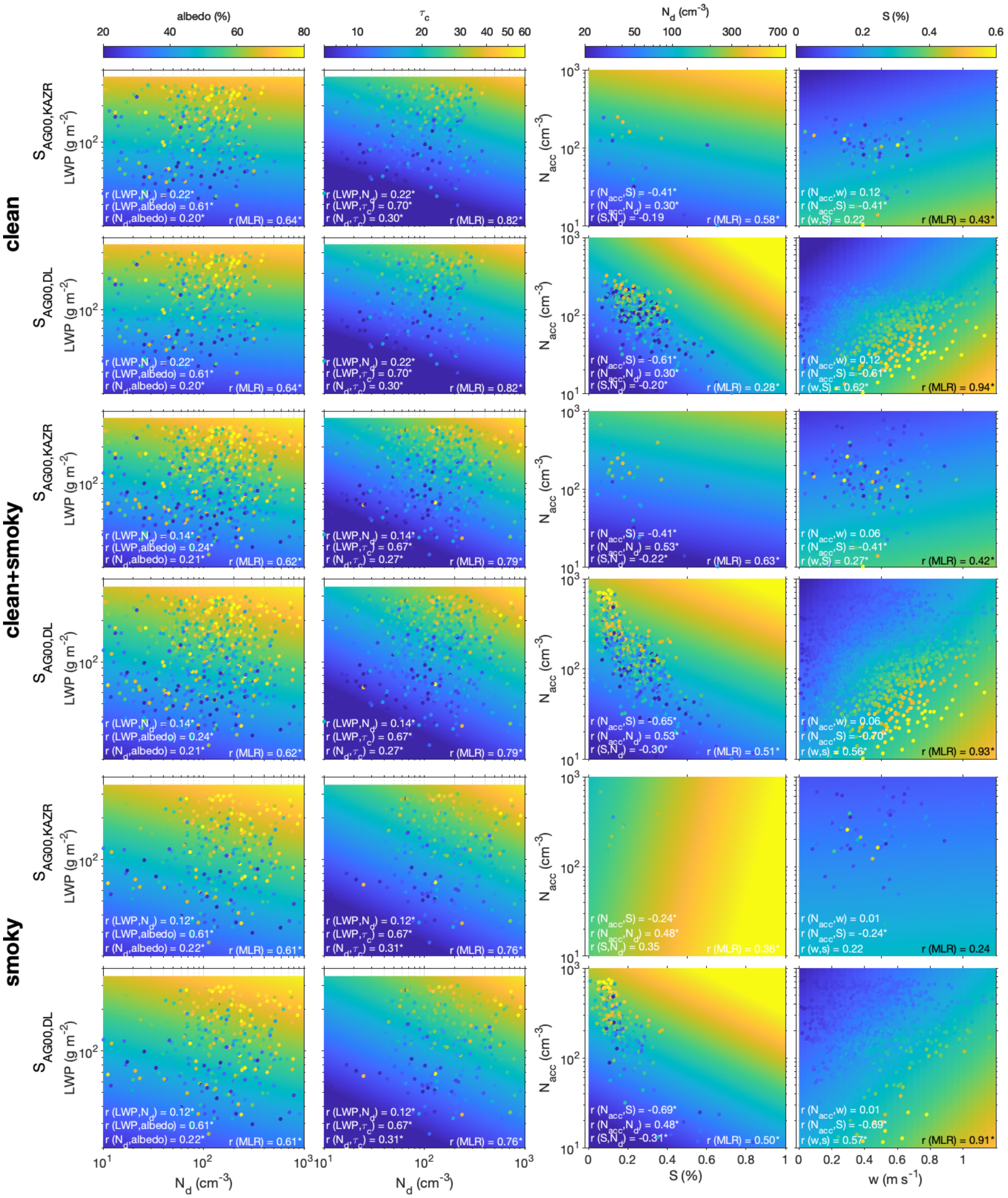

Fig. S9. Same as Fig. S8, but using parcel-based supersaturations: Multi-Mode Size-Resolving Lagrangian with lidar ($S_{AG00,DL}$) and KAZR ($S_{AG00,KAZR}$).

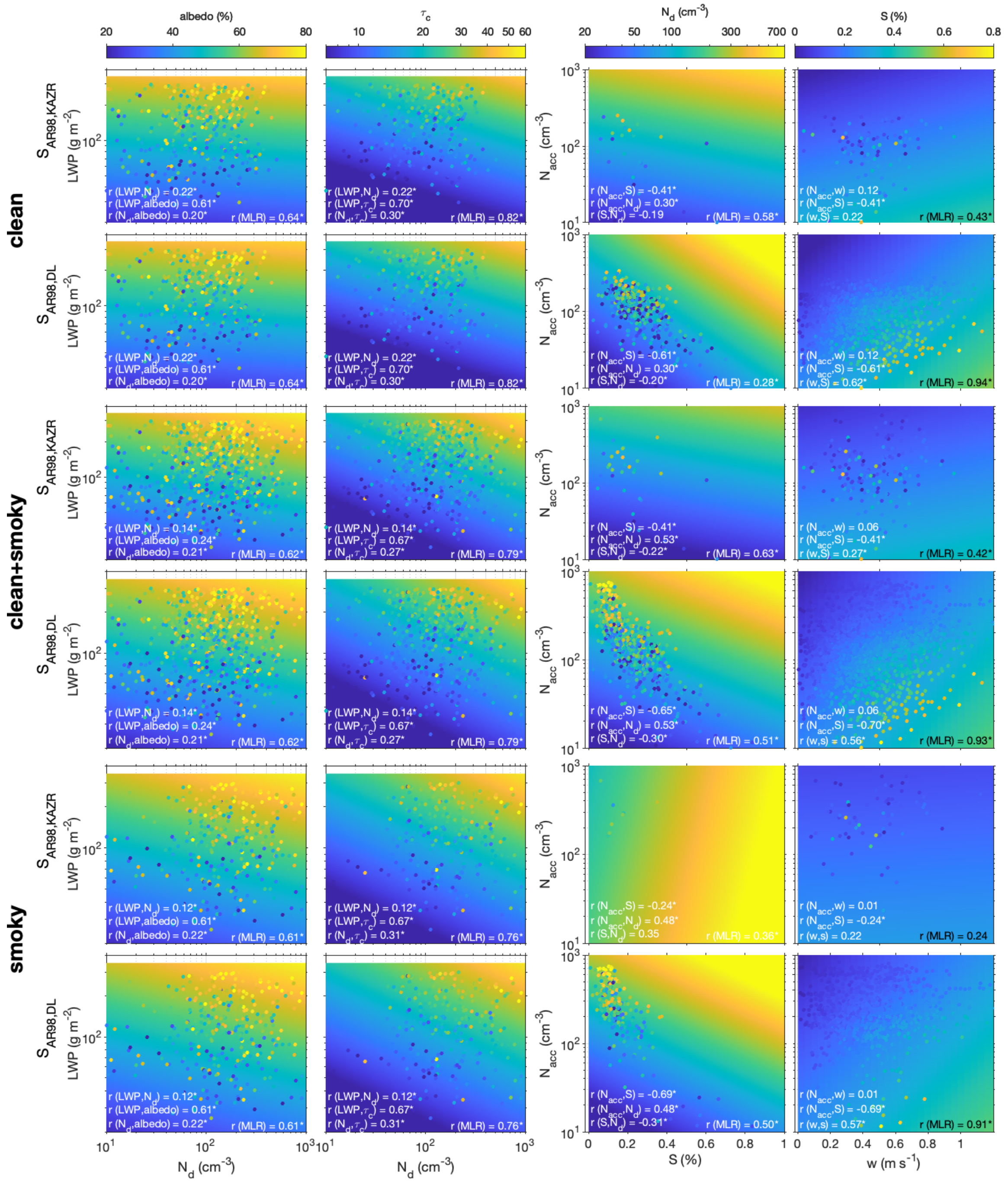

Fig. S10. Same as Fig. S9, but using the single-accumulation mode Size-Resolving Lagrangian method (S_{AR98}).

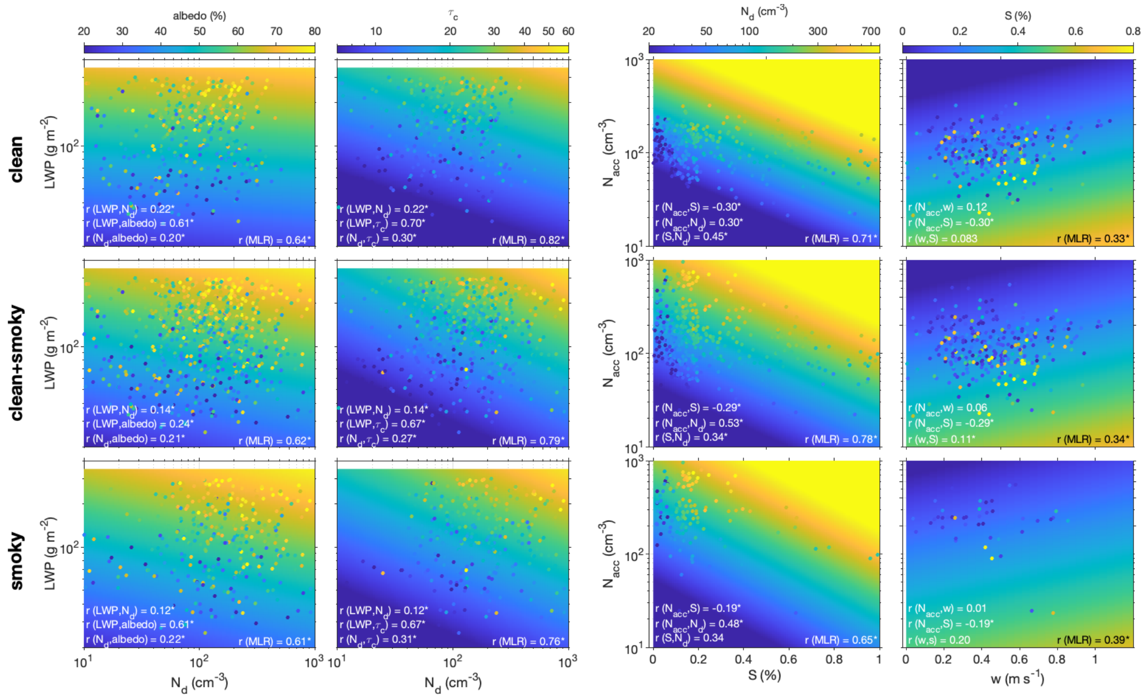

Fig. S11. Same as Fig. S8, but using cloud scene-based supersaturation (CCN and Droplet Number).

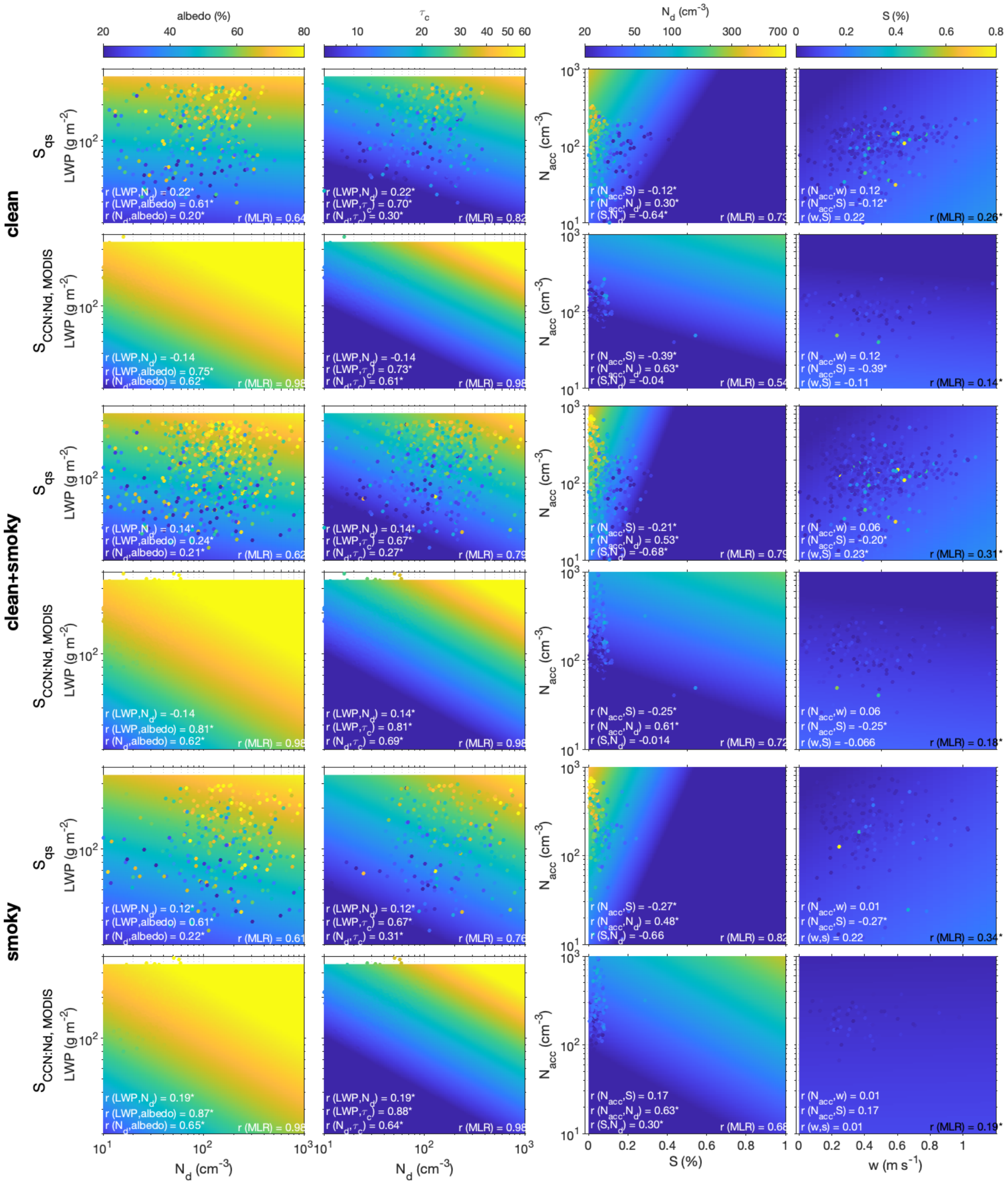

Fig. S12. Same as Fig. S10, but using the Quasi-Steady State approximation (S_{qs}) and MODIS cloud scene-based supersaturation (CCN and Droplet Number, $S_{CCN:Nd,\ MODIS}$).

Table S1. Comparison of aerosol-cloud interaction radiative forcing (RF_{ACI}) from this study and prior work.

Reference	Response	RF_{ACI} ($W\,m^{-2}$)	Method	Cloud	Aerosol
This work (cf=1.0, aerosol: tropical S. Atlantic)	S_{AG00}			N_d	N_{acc}
	Activation	-1.73±0.17	surface measurements	S_{AG00}	N_{acc}
	Competition	0.18±0.08		w	N_{acc}
	Dynamical	-0.01±0.00		α_c	N_{acc}
	Net Twomey	-1.57±0.19			
	$S_{CCN:Nd}$				
	Activation	-2.42±0.24			
	Competition	0.80±0.19			
	Dynamical	-0.02±0.004			
	Net Twomey	-1.64±0.31			
This work (c_N =0.24, aerosol: tropical S. Atlantic)	S_{AG00}				
	Activation	-0.42±0.046	surface measurements	N_d	N_{acc}
	Competition	0.043±0.031		S_{AR00}	N_{acc}
	Dynamical	-0.00390±0.00		w	N_{acc}
	Net Twomey	-0.38±0.046		α_c	N_{acc}
	$S_{CCN:Nd}$				
	Activation	-0.58±0.057			
	Competition	0.19±0.048			
	Dynamical	-0.0038±0.001			
	Net Twomey	-0.39±0.074			
(15) (cf = 1.0, aerosol: N.E. Pacific)	Twomey	-19.0 to -4.3	surface measurements	N_d	$\tau_{aerosol}$
(80) (cf = Not specified, aerosol: global)	Twomey	-0.7±0.5	satellite, GCM	N_d, α_c	$\tau_{aerosol}$,A, N_{CCN}, N_d
(77) (cf = Not specified, aerosol: global)	Twomey	-0.2±0.1	satellite, GCM	N_d, α_c	$\tau_{aerosol}$
(81) (cf = Not specified, aerosol: global)	Twomey	-0.4 (-1.01 to -0.18)	satellite, GCM	N_d, α_c	AI
(82) (cf =0.8, aerosol: global)	Twomey	-0.97±0.23 (-1.3 to -0.61)	satellite, GCM, reanalysis	N_d, α_c	SO_4^{2-} mass

(56) (cf =0.9, aerosol: global)	Twomey	-1.14 (-1.72 to -0.84)	satellite, GCM	N_d, α_c	N_{CCN}
(79) (c_N = 0.19-0.29, aerosol: global)	Twomey	-0.72* (-1.10 to -0.33)	surface measurements, satellite, GCM	N_d	$\tau_{aerosol}$
(83) (cf =0.8, aerosol: Southern Ocean)	Twomey	-0.9* (-1.2 to -0.6)	satellite	N_d	N_d
(84) (cf = Not specified, aerosol: Southern Ocean)	Twomey	-1.84* (-2.51 to -1.17)	shipboard, GCM	net low cloud radiative effect	N_{CCN}
(85) (cf = pixel average, aerosol: global)	Twomey	-0.53	satellite	N_d, cf, α_c	N_d
(86) (cf = pixel average, aerosol: Southeast Atlantic)	Twomey	-0.69 (-0.99 to -0.44)	satellite, GCM	N_d, r_e, α_c	SO_4^{2-} mass
(87) (cf = Not specified, aerosol: global)	Twomey	-0.8±0.7	satellite, GCM	N_d, cf, α_c	$\tau_{aerosol}$, N_d

S_{AG00}: Multi-Mode Size-Resolving Lagrangian supersaturation (with lidar). $S_{CCN:Nd}$: CCN & Droplet number supersaturation. c_N: effective cloud fraction for aerosol-cloud interactions (79). cf: cloud fraction. *: median value taken from upper/lower bounds. N_{acc}: accumulation-mode number concentration. N_d: droplet number. S_{AG2000}: cloud supersaturation. *w*: cloud base updraft. α_c: cloud albedo. $\tau_{aerosol}$: aerosol optical depth. AI: aerosol index. N_{CCN}: CCN number concentration.

Table S2. Average cloud microphysical properties and ACI indices from reported measurements in marine regions.

Reference	Region (type)	r_e (μm)	N_d (cm^{-3})	ACI_r	ACI_N
Clean Marine					
This study	Tropical S. Atlantic (surface)	14±4	106±87	0.12-0.2	0.47-0.59
(33)	Tropical S. Atlantic (surface)	3.81±0.6[a]	294±91	0.18±0.06	0.3±0.21
(37)	S.E. Atlantic (aircraft)		<300		
(35)	N.E. Atlantic (surface)	10.2±1.3	86±30	-0.01-0.22	
(34)	N.E. Atlantic (surface)	~12	~100		~0.7
(88)	N.E. Atlantic (surface)	5-12	<50-400	0.11-0.16	0.20-0.22
(15)	N.E Pacific (surface)	~2-20	~5->1000	0.04-0.15	0.18-0.69
(75)	N.E Pacific (aircraft)				0.8
(59)	N.E Pacific (multi-platform)				0.54-0.90
(89)	N.E. Pacific (aircraft)	~5-15	60-400	0.31-0.33	
(40)	Arctic (surface)	~5-30	~10-800	0.13-0.19	0.32-0.48
(57)	Global (satellite)				0.11 (all aerosol)
(56)	Global (satellite)				0.66 (Global) ~0.25-0.6 (South Atlantic)
Smoky/Polluted Marine					
This study	Tropical S. Atlantic (surface)	11±4	213±195	0.14-0.21	0.67-0.68
(33)	Tropical S. Atlantic (surface)	2.85±0.2[a]	611±191	0.18±0.06	0.3±0.21
(55)	S.E. Atlantic (satellite)			0.15	
(37)	S.E. Atlantic (aircraft)		~300-500		
(90)	N.W. Pacific (in-cloud)	9.9±1.5	201±90	0.2-0.34	0.2-0.4
(36)	S.E. Pacific (aircraft)	~6	~200		
(41)	Indian Ocean (multi-platform)	≤6	~ 315		0.21-0.33
(42)	E. China Sea (satellite)	~11-17		~-0.08-0.08	
(57)	S.E. Pacific (satellite)				0.62 ("high" aerosol)
(54)	Global (satellite)			0.10-0.18 (all) 0.13 (polluted)	

[a]Microphysical retrieval at cloud base using UV lidar.

Table S3. Aerosol-cloud interaction indices (ACI$_r$, ACI$_N$) controlled for meteorological regimes: free tropospheric relative humidity (FT RH), stability, and liquid water path (LWP) and the percentage of occurrence for each regime. Bold ACI indices represent values that were found to be at least weakly correlated (r>0.25) and statistically significant (p<0.05, two-tailed t test).

FT RH[a]	Dry[c]				Moist[d]			
Stability[b]	Stable[e]		Unstable[f]		Stable		Unstable	
LWP	Low[g]	High[h]	Low	High	Low	High	Low	High
Clean								
%	3	2	13	7	5	7	30	33
ACI$_r$	0.08±0.1	0.03±0.1	0.08±0.1	**0.14±0.01**	**0.33±0.1**	0.03±0.06	**0.19±0.07**	**0.12±0.04**
ACI$_N$	0.86±0.5	—	0.18±0.6	0.18±0.3	**0.92±0.4**	- 0.20±0.3	0.46±0.3	**0.62±0.1**
Smoky								
%	8	4	23	10	8	8	17	22
ACI$_r$	0.11±0.1	0.11±0.1	**0.26±0.05**	**0.21±0.08**	**0.15±0.07**	**0.28±0.06**	0.10±0.07	**0.10±0.04**
ACI$_N$	0.52±0.3	**0.77±0.2**	**0.95±0.2**	0.64±0.4	0.46±0.2	0.46±0.2	0.31±0.2	**0.91±0.13**
Clean+Smoky								
%	6	3	18	8	6	8	24	27
ACI$_r$	**0.12±0.06**	**0.13±0.06**	**0.27±0.04**	**0.18±0.04**	**0.27±0.07**	**0.16±0.4**	**0.24±0.04**	**0.18±0.02**
ACI$_N$	**0.55±0.2**	**0.46±0.1**	**0.94±0.2**	**0.51±0.2**	**0.83±0.2**	**0.32±0.2**	**0.73±0.2**	**0.75±0.08**

[a]FT RH: average radiosonde relative humidity planetary boundary layer height estimated from the ceilometer to 700 hPa. [b]Stability: lower tropospheric stability, LTS ($\theta_{700hPa} - \theta_{1000hPa}$). [c]Dry: FT RH<50%. [d]Moist: FT RH>50%. [e]Stable: LTS>17 K. [f]Stable: LTS<17 K. [g]Low LWP: 20-115 g m^{-2}. [h]High LWP: 115-300 g m^{-2}.

Table S4. ACI values separated by *w* (updraft) and LWP (liquid water path) and decomposed Twomey responses. Bold ACI represent values that were found to be at least weakly correlated (r>0.25) and statistically significant (p<0.05, two-tailed t test).

					clean	clean+ smoky	smoky
ACI	w	Low	LWP	Low	**0.18± 0.05**	**0.25± 0.03**	**0.25± 0.05**
				High	**0.18± 0.05**	**0.14± 0.02**	**0.19± 0.04**
		High	LWP	Low	**0.18± 0.05**	**0.21± 0.07**	**0.28± 0.09**
				High	**0.20± 0.04**	**0.24± 0.03**	0.077± 0.1
Twomey Responses	activation	α_c	$S_{CCN:Nd}$		1.41± 0.3	2.53± 0.2	2.59± 0.3
			S_{AG00}		0.70± 0.15	1.81± 0.18	2.10± 0.28
			S_{AR98}		0.80± 0.12	2.0± 0.20	2.21± 0.30
		τ_c	$S_{CCN:Nd}$		0.20± 0.01	0.20± 0.01	0.18± 0.01
			S_{AG00}		0.10± 0.01	0.14± 0.007	0.15± 0.01
			S_{AR98}		0.12± 0.01	0.16± 0.007	0.15± 0.01
	competition	α_c	$S_{CCN:Nd}$		-0.48± 0.2	-0.83± 0.2	-0.87± 0.4
			S_{AG00}		-0.18± 0.1	-0.19± 0.08	-0.27± 0.1
			S_{AR98}		-0.28± 0.1	-0.35± 0.01	-0.38± 0.1
		τ_c	$S_{CCN:Nd}$		-0.060± 0.01	-0.066± 0.01	-0.069± 0.03
			S_{AG00}		-0.025± 0.01	-0.015± 0.006	-0.029± 0.01
			S_{AR98}		-0.024± 0.02	-0.031± 0.009	-0.030± 0.02
	dynamical	α_c	$S_{CCN:Nd}$		0.022± 0.007	0.016± 0.004	0.01± 0.008
			S_{AG2000}		0.031± 0.01	0.017± 0.004	0.013± 0.006
			S_{AR98}		0.036± 0.01	0.021± 0.005	0.012± 0.006
		τ_c	$S_{CCN:Nd}$		0.0032± 0.0007	0.0013± 0.0003	0.0006± 0.0005
			S_{AG00}		0.0046± 0.0001	0.0014± 0.0004	0.00090± 0.0005
			S_{AR98}		0.0052± 0.0001	0.0017± 0.0004	0.00082± 0.0005
	Net		$S_{CCN:Nd}$		0.95±	1.71±	1.73±

α_c		0.4	0.3	0.6
	S_{AG00}	0.55± 0.2	1.64± 0.2	1.84± 0.4
	S_{AR98}	0.55± 0.2	1.64± 0.2	1.84± 0.4
	$S_{CCN:Nd}$	0.14± 0.02	0.14± 0.01	0.12± 0.03
τ_c				
	S_{AG00}	0.080± 0.02	0.13± 0.01	0.13± 0.02
	S_{AR98}	0.080± 0.02	0.13± 0.01	0.13± 0.02

Low w: w<0.45 m s^{-1}. High w: w>0.45 m s^{-1}. Low LWP: 20-115 g m^{-2}. High LWP: 115-300 g m^{-2}.

SI References

1. J. Dedrick *et al.*, Aerosol-Correlated Cloud Activation for Clean Conditions in the Tropical Atlantic Boundary Layer During LASIC. *Geophysical Research Letters* **51** (2024).
2. P. Zuidema *et al.*, The Ascension Island Boundary Layer in the Remote Southeast Atlantic is Often Smoky. *Geophysical Research Letters* **45**, 4456-4465 (2018).
3. J. Liu *et al.*, High summertime aerosol organic functional group concentrations from marine and seabird sources at Ross Island, Antarctica, during AWARE. *Atmospheric Chemistry and Physics* **18**, 8571-8587 (2018).
4. F. Gallo *et al.*, Identifying a regional aerosol baseline in the eastern North Atlantic using collocated measurements and a mathematical algorithm to mask high-submicron-number-concentration aerosol events. *Atmospheric Chemistry and Physics* **20**, 7553-7573 (2020).
5. A. Virkkula *et al.*, Chemical composition of boundary layer aerosol over the Atlantic Ocean and at an Antarctic site. *Atmospheric Chemistry and Physics* **6**, 3407-3421 (2006).
6. C. Kuang (2016) TSI Model 3936 Scanning Mobility Particle Spectrometer Instrument Handbook. (Brookhaven National Laboratory, Upton, NY, United States).
7. J. Uin (2016) Ultra-High Sensitivity Aerosol Spectrometer (UHSAS) instrument handbook., p 17.
8. T. Anderson *et al.*, Performance characteristics of a high-sensitivity, three-wavelength, total scatter/backscatter nephelometer. *Journal of Atmospheric and Oceanic Technology* **13**, 967-986 (1996).
9. J. Dedrick, G. Saliba, A. Williams, L. Russell, D. Lubin, Retrieval of the sea spray aerosol mode from submicron particle size distributions and supermicron scattering during LASIC. *Atmospheric Measurement Techniques* **15**, 4171-4194 (2022).
10. J. Uin (2022) Cloud Condensation Nuclei Particle Counter Instrument Handbook. (Brookhaven National Laboratory, Upton, NY, United States).
11. D. Giles *et al.*, Advancements in the Aerosol Robotic Network (AERONET) Version 3 database - automated near-real-time quality control algorithm with improved cloud screening for Sun photometer aerosol optical depth (AOD) measurements. *Atmospheric Measurement Techniques* **12**, 169-209 (2019).
12. D. Turner, Q. Min, K. Gaustad, C. Lo, D. Zhang (2014) Cloud Optical Properties from the Multifilter Shadowband Radiometer (MFRSRCLDOD). An ARM Value-Added Product. (DOE ARM Climate Research Facility).
13. Q. Min, L. Harrison, Cloud properties derived from surface MFRSR measurements and comparison with GOES results at the ARM SGP site. *Geophysical Research Letters* **23**, 1641-1644 (1996).

14. L. Riihimaki, S. McFarlane, C. Sivaraman (2021) Droplet Number Concentration Value-Added Product. (DOE ARM Climate Research Facility).
15. A. McComiskey *et al.*, An assessment of aerosol-cloud interactions in marine stratus clouds based on surface remote sensing. *Journal of Geophysical Research-Atmospheres* **114** (2009).
16. D. Zhang *et al.*, Evaluation of four ground-based retrievals of cloud droplet number concentration in marine stratocumulus with aircraft in situ measurements. *Atmospheric Measurement Techniques* **16**, 5827-5846 (2023).
17. K. Wolf *et al.*, Improvement of airborne retrievals of cloud droplet number concentration of trade wind cumulus using a synergetic approach. *Atmospheric Measurement Techniques* **12**, 1635-1658 (2019).
18. D. Rosenfeld, H. Wang, P. Rasch, The roles of cloud drop effective radius and <i>LWP</i> in determining rain properties in marine stratocumulus. *Geophysical Research Letters* **39** (2012).
19. C. Jones, C. Bretherton, D. Leon, Coupled vs. decoupled boundary layers in VOCALS-REx. *Atmospheric Chemistry and Physics* **11**, 7143-7153 (2011).
20. K. Widener, N. Bharadwaj, K. Johnson (2012) Ka-Band ARM Zenith Radar (KAZR) Handbook.
21. R. K. Newsom, C. Sivaraman, T. R. Shippert, L. D. Riihimaki (2019) Doppler Lidar Vertical Velocity Statistics Value-Added Product. (Pacific Northwest National Lab).
22. M. Jensen, ARM KAZR-ARSCL Value Added Product.
23. P. Wu, X. Dong, B. Xi, A Climatology of Marine Boundary Layer Cloud and Drizzle Properties Derived from Ground-Based Observations over the Azores. *Journal of Climate* **33**, 10133-10148 (2020).
24. H. Kalesse, P. Kollias, Climatology of High Cloud Dynamics Using Profiling ARM Doppler Radar Observations. *Journal of Climate* **26**, 6340-6359 (2013).
25. T. Fairless, M. Jensen, A. Zhou, S. E. Giangrande (2016) Interpolated Sounding and Gridded Sounding Value-Added Products. (Brookhaven National Laboratory (BNL), Upton, NY, Upton, NY (United States)).
26. R. Gelaro *et al.*, The Modern-Era Retrospective Analysis for Research and Applications, Version 2 (MERRA-2). *Journal of Climate* **30**, 5419-5454 (2017).
27. L. D. Riihimaki, K. L. Gaustad, C. N. Long (2019) Radiative Flux Analysis (RADFLUXANAL) Value-Added Product: Retrieval of Clear-Sky Broadband Radiative Fluxes and Other Derived Values. (Pacific Northwest National Laboratory).
28. J. Zhang, X. Zhou, T. Goren, G. Feingold, Albedo susceptibility of northeastern Pacific stratocumulus: the role of covarying meteorological conditions. *Atmospheric Chemistry and Physics* **22**, 861-880 (2022).

29. D. Grosvenor *et al.*, Remote Sensing of Droplet Number Concentration in Warm Clouds: A Review of the Current State of Knowledge and Perspectives. *Reviews of Geophysics* **56**, 409-453 (2018).
30. R. Bennartz, Global assessment of marine boundary layer cloud droplet number concentration from satellite. *Journal of Geophysical Research-Atmospheres* **112** (2007).
31. J. Zhang, P. Zuidema, The diurnal cycle of the smoky marine boundary layer observed during August in the remote southeast Atlantic. *Atmospheric Chemistry and Physics* **19**, 14493-14516 (2019).
32. A. Adebiyi, P. Zuidema, I. Chang, S. Burton, B. Cairns, Mid-level clouds are frequent above the southeast Atlantic stratocumulus clouds. *Atmospheric Chemistry and Physics* **20**, 11025-11043 (2020).
33. M. de Graaf *et al.*, Aerosol first indirect effect of African smoke at the cloud base ofmarine cumulus clouds over Ascension Island, southern Atlantic Ocean. *Atmospheric Chemistry and Physics* **23**, 5373-5391 (2023).
34. A. Varble *et al.*, Evaluation of liquid cloud albedo susceptibility in E3SM using coupled eastern North Atlantic surface and satellite retrievals. *Atmospheric Chemistry and Physics* **23**, 13523-13553 (2023).
35. X. Zheng *et al.*, Environmental effects on aerosol-cloud interaction in non-precipitating marine boundary layer (MBL) clouds over the eastern North Atlantic. *Atmospheric Chemistry and Physics* **22**, 335-354 (2022).
36. C. Twohy *et al.*, Impacts of aerosol particles on the microphysical and radiative properties of stratocumulus clouds over the southeast Pacific Ocean. *Atmospheric Chemistry and Physics* **13**, 2541-2562 (2013).
37. M. Kacarab *et al.*, Biomass burning aerosol as a modulator of the droplet number in the southeast Atlantic region. *Atmospheric Chemistry and Physics* **20**, 3029-3040 (2020).
38. D. Grosvenor *et al.*, Remote Sensing of Droplet Number Concentration in Warm Clouds: A Review of the Current State of Knowledge and Perspectives. *Reviews of Geophysics* **56**, 409-453 (2018).
39. M. Miller *et al.*, Observed Relationships Between Cloud Droplet Effective Radius and Biogenic Gas Concentrations in Summertime Marine Stratocumulus Over the Eastern North Atlantic. *Earth and Space Science* **9** (2022).
40. T. Garrett, C. Zhao, X. Dong, G. Mace, P. Hobbs, Effects of varying aerosol regimes on low-level Arctic stratus. *Geophysical Research Letters* **31** (2004).
41. V. Ramanathan *et al.*, Indian Ocean Experiment: An integrated analysis of the climate forcing and effects of the great Indo-Asian haze. *Journal of Geophysical Research-Atmospheres* **106**, 28371-28398 (2001).
42. P. Khatri, K. Yoshida, T. Hayasaka, Aerosol Effects on Water Cloud Properties in Different Atmospheric Regimes. *Journal of Geophysical Research-Atmospheres* **128** (2023).
43. H. Jia, X. Ma, Y. Liu, Exploring aerosol-cloud interaction using VOCALS-REx aircraft measurements. *Atmospheric Chemistry and Physics* **19**, 7955-7971 (2019).

44. X. Dong, A. Schwantes, B. Xi, P. Wu, Investigation of the marine boundary layer cloud and CCN properties under coupled and decoupled conditions over the Azores. *Journal of Geophysical Research-Atmospheres* **120**, 6179-6191 (2015).
45. P. Zuidema *et al.*, On Trade Wind Cumulus Cold Pools. *Journal of the Atmospheric Sciences* **69**, 258-280 (2012).
46. R. Wood, C. Bretherton, On the relationship between stratiform low cloud cover and lower-tropospheric stability. *Journal of Climate* **19**, 6425-6432 (2006).
47. E. Gryspeerdt *et al.*, Constraining the aerosol influence on cloud liquid water path. *Atmospheric Chemistry and Physics* **19**, 5331-5347 (2019).
48. Y. Chen, M. Christensen, G. Stephens, J. Seinfeld, Satellite-based estimate of global aerosol-cloud radiative forcing by marine warm clouds. *Nature Geoscience* **7**, 643-646 (2014).
49. S. Kirschler *et al.*, Seasonal updraft speeds change cloud droplet number concentrations in low-level clouds over the western North Atlantic. *Atmospheric Chemistry and Physics* **22**, 8299-8319 (2022).
50. J. Hudson, S. Noble, CCN and Vertical Velocity Influences on Droplet Concentrations and Supersaturations in Clean and Polluted Stratus Clouds. *Journal of the Atmospheric Sciences* **71**, 312-331 (2014).
51. V. Ghate, M. Miller, L. DiPretore, Vertical velocity structure of marine boundary layer trade wind cumulus clouds. *Journal of Geophysical Research-Atmospheres* **116** (2011).
52. R. Vogel, H. Konow, H. Schulz, P. Zuidema, A climatology of trade-wind cumulus cold pools and their link to mesoscale cloud organization. *Atmospheric Chemistry and Physics* **21**, 16609-16630 (2021).
53. M. Cadeddu, V. Ghate, M. Mech, Ground-based observations of cloud and drizzle liquid water path in stratocumulus clouds. *Atmospheric Measurement Techniques* **13**, 1485-1499 (2020).
54. P. Ma, P. Rasch, H. Chepfer, D. Winker, S. Ghan, Observational constraint on cloud susceptibility weakened by aerosol retrieval limitations. *Nature Communications* **9** (2018).
55. L. Costantino, F. Bréon, Aerosol indirect effect on warm clouds over South-East Atlantic, from co-located MODIS and CALIPSO observations. *Atmospheric Chemistry and Physics* **13**, 69-88 (2013).
56. O. Hasekamp, E. Gryspeerdt, J. Quaas, Analysis of polarimetric satellite measurements suggests stronger cooling due to aerosol-cloud interactions. *Nature Communications* **10** (2019).
57. E. Gryspeerdt *et al.*, Uncertainty in aerosol-cloud radiative forcing is driven by clean conditions. *Atmospheric Chemistry and Physics* **23**, 4115-4122 (2023).
58. B. Kim, M. Miller, S. Schwartz, Y. Liu, Q. Min, The role of adiabaticity in the aerosol first indirect effect. *Journal of Geophysical Research-Atmospheres* **113** (2008).

59. A. Sorooshian *et al.*, On the link between ocean biota emissions, aerosol, and maritime clouds: Airborne, ground, and satellite measurements off the coast of California. *Global Biogeochemical Cycles* **23** (2009).
60. J. Chen, Y. Liu, M. Zhang, Y. Peng, New understanding and quantification of the regime dependence of aerosol-cloud interaction for studying aerosol indirect effects. *Geophysical Research Letters* **43**, 1780-1787 (2016).
61. L. Costantino, F. Bréon, Analysis of aerosol-cloud interaction from multi-sensor satellite observations. *Geophysical Research Letters* **37** (2010).
62. M. Petters, S. Kreidenweis, A single parameter representation of hygroscopic growth and cloud condensation nucleus activity. *Atmospheric Chemistry and Physics* **7**, 1961-1971 (2007).
63. X. Gong *et al.*, Maximum Supersaturation in the Marine Boundary Layer Clouds Over the North Atlantic. *AGU Advances* **4** (2023).
64. L. Russell *et al.*, EASTERN PACIFIC EMITTED AEROSOL CLOUD EXPERIMENT. *Bulletin of the American Meteorological Society* **94**, 709-+ (2013).
65. J. Hudson, S. Noble, S. Tabor, Cloud supersaturations from CCN spectra Hoppel minima. *Journal of Geophysical Research-Atmospheres* **120**, 3436-3452 (2015).
66. H. Abdul-Razzak, S. Ghan, C. Rivera-Carpio, A parameterization of aerosol activation - 1. Single aerosol type. *Journal of Geophysical Research-Atmospheres* **103**, 6123-6131 (1998).
67. H. Abdul-Razzak, S. Ghan, A parameterization of aerosol activation 2. Multiple aerosol types. *Journal of Geophysical Research-Atmospheres* **105**, 6837-6844 (2000).
68. A. Gettelman *et al.*, The Single Column Atmosphere Model Version 6 (SCAM6): Not a Scam but a Tool for Model Evaluation and Development. *Journal of Advances in Modeling Earth Systems* **11**, 1381-1401 (2019).
69. S. Bauer *et al.*, Historical (1850-2014) Aerosol Evolution and Role on Climate Forcing Using the GISS ModelE2.1 Contribution to CMIP6. *Journal of Advances in Modeling Earth Systems* **12** (2020).
70. H. Kawai *et al.*, Significant improvement of cloud representation in the global climate model MRI-ESM2. *Geoscientific Model Development* **12**, 2875-2897 (2019).
71. D. Lamb, J. Verlinde, *Physics and Chemistry of Clouds* (Cambridge University PRess, 2011).
72. I. Gultepe, G. Isaac, Aircraft observations of cloud droplet number concentration: Implications for climate studies. *Quarterly Journal of the Royal Meteorological Society* **130**, 2377-2390 (2004).
73. F. Yang *et al.*, A new approach to estimate supersaturation fluctuations in stratocumulus cloud using ground-based remote-sensing measurements. *Atmospheric Measurement Techniques* **12**, 5817-5828 (2019).
74. A. S. Williams *et al.*, Aerosol Size Distribution Properties Associated with Cold-Air Outbreaks in the Norwegian Arctic. *EGUsphere Discussions* (2024).

75. B. Schulze *et al.*, Characterization of Aerosol Hygroscopicity Over the Northeast Pacific Ocean: Impacts on Prediction of CCN and Stratocumulus Cloud Droplet Number Concentrations. *Earth and Space Science* **7** (2020).
76. M. Gaetani, B. Pohl, M. Castro, C. Flamant, P. Formenti, A weather regime characterisation of winter biomass aerosol transport from southern Africa. *Atmospheric Chemistry and Physics* **21**, 16575-16591 (2021).
77. J. Quaas, O. Boucher, N. Bellouin, S. Kinne, Satellite-based estimate of the direct and indirect aerosol climate forcing. *Journal of Geophysical Research-Atmospheres* **113** (2008).
78. L. Riihimaki, D. Zhang, K. Gaustad, [Dataset] radflux1long. USDOE Office of Science (SC), Biological and Environmental Research (BER).
79. N. Bellouin *et al.*, Bounding Global Aerosol Radiative Forcing of Climate Change. *Reviews of Geophysics* **58** (2020).
80. P. Forster *et al.* (2021) The Earth's Energy Budget, Climate Feedbacks, and Climate Sensitivity, in: Climate Change 2021: The Physical Science Basis. Contribution of Working Group I to the Sixth Assessment Report of the Intergovernmental Panel on Climate Change. eds V. Masson-Delmotte *et al.*
81. E. Gryspeerdt *et al.*, Constraining the instantaneous aerosol influence on cloud albedo. *Proceedings of the National Academy of Sciences of the United States of America* **114**, 4899-4904 (2017).
82. D. Mccoy *et al.*, The global aerosol-cloud first indirect effect estimated using MODIS, MERRA, and AeroCom. *Journal of Geophysical Research-Atmospheres* **122**, 1779-1796 (2017).
83. I. McCoy *et al.*, The hemispheric contrast in cloud microphysical properties constrains aerosol forcing. *Proceedings of the National Academy of Sciences of the United States of America* **117**, 18998-19006 (2020).
84. L. Regayre *et al.*, The value of remote marine aerosol measurements for constraining radiative forcing uncertainty. *Atmospheric Chemistry and Physics* **20**, 10063-10072 (2020).
85. V. Toll, M. Christensen, J. Quaas, N. Bellouin, Weak average liquid-cloud-water response to anthropogenic aerosols. *Nature* **572**, 51-+ (2019).
86. M. Diamond, H. Director, R. Eastman, A. Possner, R. Wood, Substantial Cloud Brightening From Shipping in Subtropical Low Clouds. *Agu Advances* **1** (2020).
87. D. Arndt *et al.*, STATE OF THE CLIMATE IN 2017. *Bulletin of the American Meteorological Society* **99**, S1-S310 (2018).
88. K. Mansour *et al.*, Phytoplankton Impact on Marine Cloud Microphysical Properties Over the Northeast Atlantic Ocean. *Journal of Geophysical Research-Atmospheres* **127** (2022).
89. C. Twohy *et al.*, Evaluation of the aerosol indirect effect in marine stratocumulus clouds: Droplet number, size, liquid water path, and radiative impact. *Journal of Geophysical Research-Atmospheres* **110** (2005).

90. R. Misumi *et al.*, Classification of aerosol-cloud interaction regimes over Tokyo. *Atmospheric Research* **272** (2022).
